# Polyoxygenated Klysimplexane- and Eunicellin-Based Diterpenoids from the Gorgonian *Briareum violaceum*

**DOI:** 10.3390/molecules26113276

**Published:** 2021-05-28

**Authors:** Atallah F. Ahmed, Yang Cheng, Chang-Feng Dai, Jyh-Horng Sheu

**Affiliations:** 1 Department of Pharmacognosy, College of Pharmacy, King Saud University, Riyadh 11451, Saudi Arabia; afahmed@ksu.edu.sa; 2 Department of Pharmacognosy, Faculty of Pharmacy, Mansoura University, Mansoura 35516, Egypt; 3 Department of Marine Biotechnology and Resources, National Sun Yat-sen University, Kaohsiung 804, Taiwan; jack1991106@yahoo.com.tw; 4 Institute of Oceanography, National Taiwan University, Taipei 112, Taiwan; corallab@ntu.edu.tw; 5 Graduate Institute of Natural Products, Kaohsiung Medical University, Kaohsiung 807, Taiwan; 6 Frontier Center for Ocean Science and Technology, National Sun Yat-sen University, Kaohsiung 804, Taiwan; 7 Department of Medical Research, China Medical University Hospital, China Medical University, Taichung 404, Taiwan

**Keywords:** *Briareum violaceum*, klysimplexane, briarol, eunicellin, gorgonian

## Abstract

Three new polyoxygenated diterpenoids with a rare 4-isopropyl-1,5,8a-trimethylperhydrophenanthrane structure of the klysimplexane skeleton, briarols A‒C (1‒3), and one eunicellin-based diterpenoid, briarol D (4), were isolated from *Briareum violaceum*, a gorgonian inhabiting Taiwanese waters. The chemical structures of these compounds were determined by employing extensive analyses of NMR and high-resolution electrospray ionization mass spectrometry (HRESIMS) data. Metabolites 1‒3 were found to possess the rarely found skeleton of the diterpenoid klysimplexin T. All isolated compounds showed very weak cytotoxic activity against the growth of three cancer cell lines. A plausible biosynthetic pathway for briarols A‒C from the coexisting eunicellin diterpenoid briarol D (4) was postulated.

## 1. Introduction

**Figure 1:**
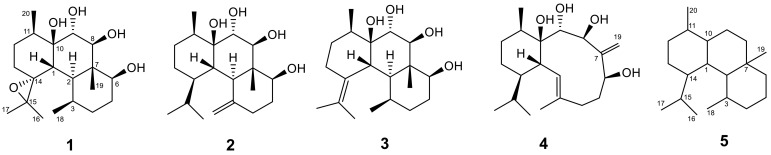
Structures of new diterpenoids isolated from *B. violaceum* (**1**–**4**) and the klysimplexane skeleton (**5**).

Gorgonian corals belonging to genus *Briareum* (Cnidaria, Octocorallia, Briareidae) inhabiting the western Pacific Ocean and Caribbean waters have been found to be a rich source of diterpenoids [1,2] possessing fused bicarbocyclic structures of briarane [3,4,5,6,7], eunicellin [8,9,10,11], and asbestinane [12,13,14] types, in addition to cembranoids [15,16]. Many of these metabolites exhibit a wide range of bioactivities, including anti-inflammatory [3,11,17,18,19,20], cytotoxic [14,21,22,23], antiviral [14,21,24], antimalarial [8], antimicrobial [14], and analgesic [20] activities. Our previous study on the chemical constituents of *Briareum violaceum* afforded the isolation of briarellins (2,9:3,16-diepoxyeunicellins), which were shown to possess interesting structures generated from intramolecular cyclization of corresponding cembranoids [10]. In our efforts to discover new natural products from marine organisms, a continuous chemical investigation of *B. violaceum* was carried out. The present study led to the discovery of four new diterpenoids. Three of them, briarols A‒C (1‒3), were identified as compounds of a rare (4-isopropyl-1,5,8a-trimethylperhydrophenanthrane) skeleton, which was discovered for only one time as klysimplexin T in 2011 [25] and is herein denominated as the klysimplexane skeleton (Figure 1). The structure elucidation of the new metabolites was performed by extensive spectroscopic analyses, including two-dimensional (2D) NMR correlation and high-resolution electrospray ionization mass spectroscopy (HRESIMS) analyses. A plausible biosynthetic pathway was suggested and the cytotoxicity of the new compounds was evaluated.

## 2. Results and Discussion

The lyophilized organism was extracted with ethyl acetate (EtOAc) followed by chromatographic fractionation of solvent-free extract on silica (Si) gel. Fractions showing ^1^H NMR signals characteristic of polyoxygenated terpenoids were separated mainly by a reverse-phase (RP) column and high-performance liquid chromatography (RP-HPLC), yielding diterpenoids 1‒4 (Figure 1). The spectra of these compounds are given in the Supplementary Materials (Figures S4–S40). The IR absorption bands at ν_max_ 3413–3464 cm^−1^ and the four ^13^C NMR signals, resonating at the region of δ_C_ 70.7 to 81.9 ppm, disclosed the multi-hydroxylated pattern of the isolated compounds.

**Figure 2:**
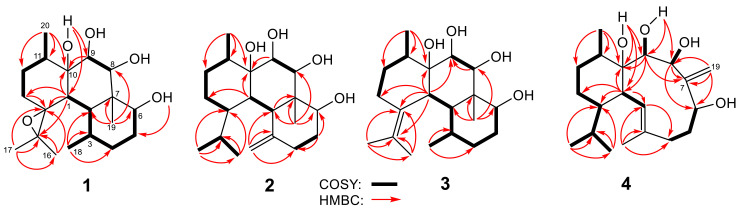
Key proton homonuclear correlation spectroscopy (^1^H-^1^H COSY) and heteronuclear multiple bond correlation (HMBC) correlations for (**1**–**4**).

Briarol A (**1**) was obtained as a white powder with an optical rotation of ${\left[\alpha \right]}_{D}^{25}$ = −101.9 (*c* 0.24, CHCl_3_). The sodiated ion peak at *m/z* 377.2300 [M + Na]^+^ in the HRESIMS established a molecular formula of C_20_H_34_O_5_ for **1**, appropriate for four degrees of unsaturation. The IR absorption at ν_max_ 3430 cm^−1^ revealed the presence of hydroxy functionality. As the ^1^H NMR spectrum, measured in CDCl_3_, showed nine overlapped proton signals at δ_H_ 1.50–1.80 ppm, we remeasured **1** in C_6_D_6_ and acetone-*d_6_* to allow better signal resolution and to facilitate integrated 2D NMR correlation analyses (Figure 2 and Figures S2–S19). The ^13^C NMR spectrum of **1**, combined with distortionless enhancement by polarization transfer (DEPT) and heteronuclear single quantum correlation (HSQC) spectra, displayed 20 sp^3^-hybridized carbon signals (δ_C_ 10.9–81.9 ppm) assignable for 5 methyl, 4 methylene, 7 methine, and 4 quaternary carbons (Table 1). Therefore, the four degrees of unsaturation identified metabolite **1** as a tetracyclic diterpenoid. Analyzing proton homonuclear correlation spectroscopy (^1^H-^1^H COSY) correlations revealed the presence of three partial structures of consecutive proton systems extending from H-1 and H_3_-18 to H-6 through to H-3, from H-8 to H-9, and from H_3_-20 to H_2_-13 through H-11 (Figure 2). The *^2^J_CH_* and *^3^J_CH_* correlations, as determined by the heteronuclear multiple bond correlation (HMBC) experiments, established the connectivities of the partial structures, and hence the 6-6-6 tricarbocyclic framework of **1** (Figure 2). The four most downfield-shifted carbon signals in the ^13^C NMR spectrum (δ_C_ 78.9–81.9) were attributable to four hydroxy-bearing carbons. Thus, the remaining oxygen atom in the molecular formula of **1** together with the two upfield-shifted oxycarbons (δ_C_ 70.9, C and 64.2, C) suggested the presence of a tetrasubstituted epoxy ring. Four 1H singlets (δ_H_ 4.54, 4.12, 2.94, and 2.32; Table 2), lacking HSQC correlations, were assigned to the protons of four hydroxy groups. Two protons of these (δ_H_ 4.12 and 4.54), exhibiting HMBC correlations with C-5 (δ_C_ 25.4, CH_2_) and C-9 (δ_C_ 79.8, CH)/C-1 (δ_C_ 43.5, CH), were recognized as 6-OH and 10-OH, respectively (Figure 2). The HMBC correlations from both H-1 and CH_3_-20 to C-10 (δ_C_ 78.9, C) confirmed the presence of a hydroxy group at C-10. Moreover, the HMBC correlations observed for H_3_-19 (δ_H_ 1.09 3H, s) with the oxymethine carbons C-6 and C-8 together with the ^1^H-^1^H COSY correlation H-8/H-9 are indicative of the hydroxy groups at C-8 and C-9, respectively (Figure 2). Furthermore, the long-range connectivities from the protons of the tertiary methyls H_3_-16 and H_3_-17 (δ_H_ 1.08 and 0.97, each 3H, s) and from the angular methine proton H-1 (δ_H_ 1.64, d, *J =* 12.0 Hz) to the oxycarbons C-14 (δ_C_ 70.9) and C-15 (δ_C_ 64.2) placed the epoxy group at C-14/C-15. The above findings and other detailed 2D NMR correlation analyses unambiguously established the planar structure of **1** (Figure 2).

**Figure 3:**
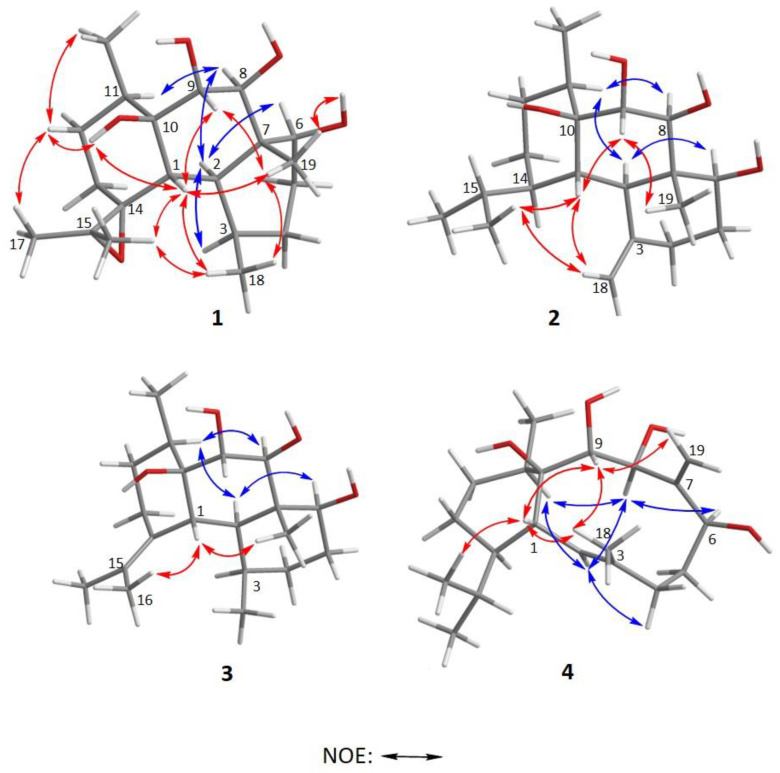
Selected nuclear Overhauser effect (NOE) correlations for (**1**–**4**).

The relative configurations of the 10 chiral carbons in **1** were mostly deduced by examining nuclear Overhauser effect (NOE) correlations (Figure 3). The large *^3^J_H–H_* value of the ring juncture protons H-1 and H-2 (12.0 Hz) must be due to *anti* orientation of the two axial protons, which were assumed to be on the β- and α-faces of the molecule, respectively. Therefore, the key NOE interactions of H-1 with H_3_-18, H_3_-19, H-9, and 10-OH (red-colored arrows) revealed these protons to be cofacial, indicating the α-oriented hydroxy group at C-9 and hence the S*,R*,S*,R*, R*-configurations at C-1, C-3, C-7, C-9, and C-10, respectively. Consequently, the NOE correlations found for H-3 with H-2, H-2 with H-6 and H-8, and H-8 with H-11 (blue-colored arrows) designated the S*,S*,S*,R*-configurations at C-2, C-6, C-8, and C-11, respectively. However, the NOE interaction of H-1 with H_3_-16 could not be used for effective elucidation of the relative configuration at C-14. Fortunately, the NOE correlations for H_3_-20/H-12β and H-12β/H_3_-17 were observed in the Nuclear Overhauser Effect Spectroscopy NOESY spectra of **1**, measured in both CDCl_3_ and acetone-*d_6_*, and established the α-orientation of the 14,15-epoxy group. Therefore, briarol A (**1**) could be defined as (1S*,2S*,3R*,6S*,7S*,8S*,9R*,10R*,11R*,14R*)-14:15-epoxy-klysimplexan-6,8,9,10-tetrol.

Briarol B (2) was obtained as a white powder. It possessed the molecular formula of C_20_H_34_O_4_ as indicated by the adduct ion peak at *m/z* 361.2348 [M + Na]^+^ in its HREIMS, with 16 mass units fewer than that of 1. A comparison of the ^13^C and ^1^H NMR data of 2 with those of 1 (Table 1 and Table 2, respectively) revealed the presence of another klysimplexane-based metabolite. However, the NMR spectroscopic data of 2 showed the appearance of an olefinic double bond (δ_C_ 144.1, C and 111.8, CH_2_; δ_H_ 5.02 and 4.81, each 1H, s) and the absence of the epoxy group. Thus, the two oxycarbons (δ_C_ 70.9 and 64.2, each C) and the methylated methine carbons (δ_C_ 17.2, CH_3_ and 28.5, CH) in 1 were replaced by the carbons of two methines (δ_C_ 43.9 and 28.5, each CH) and a 1,1-disubstituted double bond in 2, respectively. These carbons were then assigned as C-14, C-15, C-3, and C-18, respectively, from the 2D NMR correlation analyses of 2 (Figure 2). Therefore, the gross structure of compound 2 was recognized as klysimplexan-3(18)-en-6,8,9,10-tetrol. The investigation of NOE correlations of 2 (Figure 3) resulted in the same relative configurations at C-1, C-6, C-7, C-8, C-9, C-10, and C-11 as those of 1. Furthermore, the NOE interactions found for the β-oriented H-1 (δ_H_ 2.08, m) with H_3_-16 (δ_H_ 0.92, 3H, d, *J =* 6.5 Hz), H_3_-16 with one of the exo-methylene protons (δ_H_ 4.81, s, H-18b), and H-18b with H-1 favored the β-orientation for the 14-isopropyl group and thus the R* configuration at C-14. These findings together with detailed 2D NMR correlations (Figure 2 and Figure 3) unambiguously established compound 2 as (1S*,2R*,6S*,7S*,8S*,9R*,10R*,11R*,14R*)- klysimplexan-3(18)-en-6,8,9,10-tetrol.

Briarol C (3) was also isolated as a white powder that gave a pseudomolecular ion peak at 361.2347 [M + Na]^+^ in the HRESIMS, consistent with the molecular formula C_20_H_34_O_4_ and four degrees of unsaturation, as with 2. The ^13^C NMR spectroscopic data of 3 were found to be in accordance with those of 1 from C-1 to C-13 and C-16 to C-20, except for the presence of a tetrasubstituted double bond (δ_C_ 128.3 and 128.0, each C) instead of the tetrasubstituted epoxy group in 1 (Table 1). Thus, the *^2^J_CH_* and *^3^J_CH_* correlations displayed by the two olefinic methyl protons (δ_H_ 1.81 and 1.74, each 3H, s) with the olefinic carbons (δ_C_ 128.3 and 128.0, each C), which in turn were correlated with H-1 (δ_H_ 2.81, d, *J =* 7.5 Hz), confirmed the presence of a 14,15-double bond (Figure 2). The relative configuration of 2 was deduced from NOESY correlations, as illustrated in Figure 3. Furthermore, it was found that the ^13^C NMR chemical shifts of C-1 to C-11 in 1 and ^1^H NMR data of H-6, H-8, and H-9 in 1 and 2 (Table 2) were analogous to those of 3, reflecting the same β-orientation for H-1, 6-OH, 8-OH, 10-OH, H_3_-18, H_3_-19, and H_3_-20, and the α-orientation for H-2 and 9-OH. Therefore, compound 3 was clearly identified as (1R*,2S*,3R*,6S*,7S*,8S*,9R*,10R*,11R*)-klysimplexan-14(15)-en-6,8,9,10-tetrol.

Briarol D (4) was obtained as a white powder and gave a sodiated ion peak at *m/z* 361.2349 [M + Na]^+^ by HREIMS, appropriate for a molecular formula of C_20_H_34_O_4_ and four degrees of unsaturation. The ^13^C NMR and DEPT spectra indicated the presence of 20 carbon signals (Table 1) corresponding to 4 methyls, 5 methylenes (including 1 exomethylene), 8 methines (including 1 olefinic and 3 oxymethines), and 3 quaternary carbons (2 olefinic and 1 oxycarbon) of a diterpenoid. The NMR spectroscopic data (Table 1 and Table 2) revealed the presence of a trisubstituted [δ_C_ 125.5, CH, 137.7, C; δ_H_ 5.35 (d, *J* = 10.0 Hz)] and an 1,1-substituted [δ_C_ 162.8, C, 113.8, CH_2_ and δ_H_ 5.43, 5.37 (each d, *J* = 1.2 Hz)] double bond. The remaining two degrees of unsaturation were thus attributed to a bicyclic structure for 4. This was further substantiated by the NMR data comparison of 4 with those of 1–3, which showed the substitution of one ring-juncture methine (2-CH) with an olefinic methine [δ_H_/δ_C_ 5.35 (d, *J* = 10.0 Hz)/125.5, CH] in 4. However, the ^1^H and ^13^C NMR data pointed out the presence of three hydroxy-bearing methines [δ_H_/δ_C_ 4.21 (d, *J* = 4.5 Hz, H-8)/70.7; 4.08 (d, *J* = 7.5 Hz, H-6)/76.2; and 3.46 (d, *J* = 5.5 Hz, H-9)/80.9], as in the case of compounds 1‒3. Moreover, two protons resonating at δ_H_ 4.26 (d, *J* = 4.5 Hz) and 4.00 (d, *J* = 5.5 Hz) exhibited COSY correlations with H-8 and H-9 due to 8-OH and 9-OH, respectively. The gross structure of 4 as a eunicellin-derived diterpenoid [26], including the positions of the two olefinic bonds and the four hydroxy groups, was further resolved by the study of the long-range proton–carbon correlations (Figure 2). In particular, the HMBC correlations found from the only available ring-juncture proton (δ_H_ 2.52, dd, *J* = 10.0, 3.0 Hz, H-1) to C-2 (δ_C_ 125.5, CH) and C-10 (δ_C_ 78.3, C), from the olefinic methyl protons (δ_H_ 1.57, s, H_3_-18) to C-2, C-3 (δ_C_ 137.7, C), and C-4 (δ_C_ 38.1, CH_2_) and from the exomethylene protons (δ_H_ 5.43, 5.37, each d, *J* = 1.2 Hz, H_2_-19) to C-6 (δ_C_ 76.2, CH), C-7 (δ_C_ 162.8, C), and C-8 (δ_C_ 70.7, CH), positioned the trisubstituted and 1,1-disubstituted double bonds at C-2/C-3 and C-7, respectively. Based on the above findings and detailed 2D NMR correlations (Figure 2), the molecular framework of 4 was established.

An inspection of NOESY correlations (Figure 3) enabled us to assign the relative configurations of the seven chiral carbons C-1, C-6, C-8, C-9, C-10, C-11, and C-14 in 4. The NOE correlations observed for the β-oriented ring-juncture proton H-1 with the protons of the 9-oxymethine and one of the 14-isopropyl methyls reflected the α-orientation of H-14 and 9-OH. Furthermore, the NOE observed for H-1/H_3_-18 and H-2/H-4 combined with upfield chemical shift (δ_C_ < 20 ppm) observed for C-18 (δ_C_ 18.3 ppm) determined the *E*-geometry of the olefinic bond [27] at C-2/C-3. This finding placed the olefinic H-2 on the α-face of the molecule. Consequently, the NOE interactions found for H-2 with H-8 and H-8 with both H-6 and H-11 revealed the β-orientation for H-6, H-8, H-10, and H_3_-20. Compound 4 was thus unambiguously identified as (1S*,2*E*,6S*,8S*,9R*,10R*,11R*)-eunicellin-2,7(19)-dien-6,8,9,10-tetrol.

**Scheme 1:**
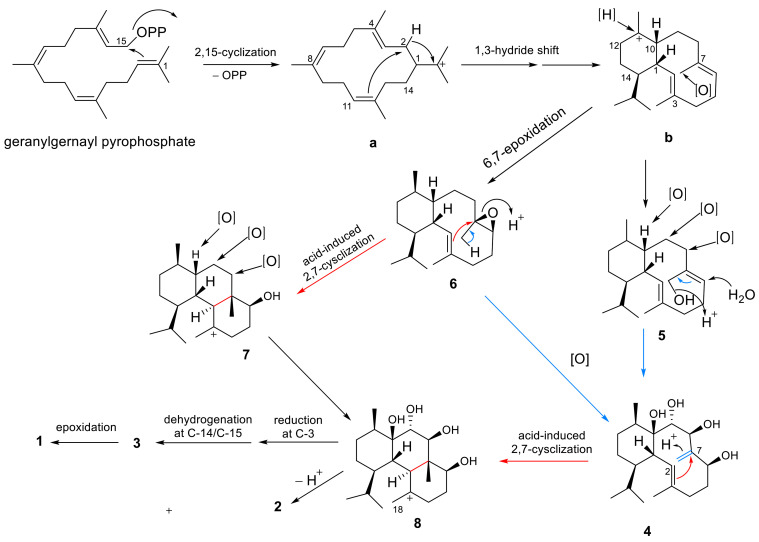
A plausible biosynthetic pathway for 1–4.

Based on the above discoveries, it is proposed that compounds 1–4 can be derived from the common eunicellin intermediate (b) after the 2,11-cyclization and 1,3-hydride shift of a cembranoid cation (a). Oxidation of CH_2_-8, CH_2_-9, CH-10, and CH_3_-18 followed by acid-catalyzed hydroxylation at the olefinic C-6 with a subsequent formation of an exomethylene at C-7 in the intermediate 5 yields 4. Furthermore, the 6,7-epoxidation for the intermediate b gives 6 as the intermediate of metabolite 4 and the tricarbocyclic carbonium ion 7. Both 4 and 7 could be further converted into carbonium ion 8, as shown in Scheme 1. Deprotonation at C-18 in 8 can produce 2, while reduction at C-3 and dehydrogenation at C-14/C-15 in 8 gives 3. Subsequently, the epoxidation of the olefinic double bond in metabolite 3 affords 1 (Scheme 1). To the best of our knowledge, the biosynthesis of the klysimplexane- and eunicellin-type diterpenoids is limited to marine invertebrates, and there are no analogous structures in terrestrial natural products.

The in vitro cytotoxicity of the new diterpenoid metabolites (1‒4) was assessed against the cancer cell lines of human colon cholangiocellular carcinoma (HuCC-T1), human colon carcinoma (HT-29), and human colon adenocarcinoma (DLD-1). The results showed that all compounds only exhibited very weak cytotoxicity against the tested cancer cells, with the IC_50_ values ranging from 220.75 to 238.88 μM as compared to doxorubicin hydrochloride (IC_50_ 1.38 to 2.24 μM). Because of the low yield (< 2.5 mg) and the consumption of the isolated metabolites in measurements of spectroscopic data and cytotoxicity, we suggest that further investigation on other biological activities should be carried out once these tetradroxylated diterpenoid molecules, in particular those with the rare klysimplexane skeleton, can be obtained in sufficient quantities.

## 3. Materials and Methods

### 3.1. General Experimental Procedures

IR spectra and optical rotations were measured on JASCO FT/IR-4100 spectrophotometer and JASCO P-1020 polarimeter (JASCO Corporation, Tokyo, Japan), respectively. LRESIMS and HRESIMS spectra were measured on Bruker APEX II mass spectrometer (Bruker, Bremen, Germany). ^1^H and ^13^C NMR spectra were measured on Varian Unity INOVA 600 FT-NMR (or 500 or 400 FT-NMR) instruments (Varian Inc., Palo Alto, CA, USA) at 600 MHz (or 500 or 400 MHz) for ^1^H and 150 MHz (or 125 or 100 MHz) for ^13^C in CDCl_3_ or CD_3_OD or acetone-*d_6_*. Silica (Si) gel (230–400 mesh) (Merck, Darmstadt, Germany) and C18 reverse-phase Si gel (RP-18; 40–63 µM) (Parc-Technologique Blvd, Quebec, Canada) were used for column chromatography. Thin-layer chromatography (TLC) analyses were achieved using precoated Si gel (Kieselgel 60 F-254, 0.2 mm) plates (Merck, Darmstadt, Germany). Further purification and the separation of compounds were performed by reverse-phase high-performance liquid chromatography (RP-HPLC) on a Hitachi L-2455 HPLC instrument with a Supelco C18 column (250 × 21.2 mm, 5 μm) (Supelco Inc., Bellefonte, PA, USA).

### 3.2. Animal Material

The soft coral *B. violaceum* was collected from Jihui Fish Port, Taitung, Taiwan, identified, and extracted as described before [10]. A voucher specimen was taken and deposited at the Department of Marine Biotechnology and Resources, National Sun Yat-sen (NSYSU) University, Kaohsiung.

### 3.3. Extraction and Isolation

The lyophilized bodies of soft coral (500 g, wet weight) were crushed and extracted with EtOAc. The EtOAc extract (3.9 g) was fractionated with Si gel column chromatography (CC) using EtOAc-hexane (0:100 to 100:0, gradient). Polar fractions eluted with EtOAc-hexane (10:1), which showed the diagnostic ^1^H NMR (methyl and oxymethine) signals of polyoxygenated terpenoids, were combined and subfractionated on Si gel CC using acetone-hexane (1:2.5), affording the subfractions F1 and F5. Subfraction F4 was separated on RP-18 Si gel CC using acetyl nitrite (CH_3_CN)-H_2_O (1.5:1 then 1.2:1) to give compounds **2** (1.5 mg), **3** (2.0 mg), and **4** (2.2 mg), respectively. Compound **1** (2.4 mg) was obtained from subfraction F5 with a 3-step purification process with RP-18 Si gel CC using MeOH-H_2_O (1.5:1 then 5:1), RP-HPLC using CH_3_CN-H_2_O (1:2), and then on Si gel CC using acetone-hexane (1:5).

Briarol A (1). White powder; ${\left[\alpha \right]}_{D}^{25}$ −101.9 (c 0.24, CHCl_3_); IR (neat) ν_max_ 3430, 2927, 2853, and 1382 cm^−1^; ^13^C NMR (100 MHz, C_6_D_6_) and ^1^H NMR (400 MHz, C_6_D_6_). See Table 1 and Table 2, respectively. ^13^C NMR (100 MHz, CDCl_3_) δ_C_ 81.3 (CH, C-6), 79.3 (CH, C-8), 78.7 (CH, C-9), 78.3 (C, C-10), 70.4 (C, C-14), 64.1 (C, C-15), 43.7 (C, C-7), 43.7 (CH, C-2), 42.6 (CH, C-1), 32.0 (CH, C-11), 31.5 (CH_2_, C-4), 29.6 (CH_2_, C-12), 29.6 (CH_3_, C-17), 27.6 (CH, C-3), 25.1 (CH_2_, C-13), 24.2 (CH_2_, C-5), 23.2 (CH_3_, C-16), 17.0 (CH_3_, C-20), 16.6 (CH_3_, C-18), 9.9 (CH_3_, C-19); ^1^H NMR (400 MHz, CDCl_3_) δ_H_ 4.33, 3.94, 3.23, and 2.65 (each 1H, br s, 6-OH, 8-OH, 9-OH, and 10-OH), 3.64 (1H, d, *J* = 11.2 Hz, H-8), 3.61 (1H, dd, *J* = 10.4, 5.2 Hz, H-6), 3.56 (1H, d, *J* = 11.2 Hz, H-9), 2.16 (1H, m, H-11), 1.79 (1H, d, *J* = 6.0 Hz, H-1), 1.73 (1H, m, H-3), 1.72 (1H, m, H-5β), 1.69 (1H, m, H-12β), 1.68 (1H, m, H-5α), 1.67 (1H, m, H-13β), 1.56 (2H, m, H_2_-4), 1.55 (1H, m, H-2), 1.54 (1H, m, H-12α), 1.45 (3H, s, H_3_-16), 1.39 (1H, m, H-13α), 1.33 (3H, s, H_3_-17), 1.15 (3H, d, *J* = 6.4 Hz, H_3_-20), 1.06 (3H, s, H_3_-19), 0.97 (3H, d, *J* = 7.6 Hz, H_3_-18); ^13^C NMR (100 MHz, acetone-d_6_) δ_C_ 81.9 (CH, C-6), 80.2 (CH, C-8), 79.0 (CH, C-9), 78.6 (C, C-10), 70.0 (C, C-14), 64.2 (C, C-15), 44.2 (C, C-7), 44.0 (CH, C-2), 43.5 (CH, C-1), 32.4 (CH, C-11), 31.7 (CH_2_, C-4), 30.5 (CH_2_, C-12), 28.2 (CH, C-3), 25.6 (CH_2_, C-13), 25.1 (CH_2_, C-5), 23.2 (CH_3_, C-16), 20.3 (CH_3_, C-17), 17.5 (CH_3_, C-20), 16.8 (CH_3_, C-18), 10.0 (CH_3_, C-19; ^1^H NMR (400 MHz, acetone-d_6_) δ_H_ 4.30 and 3.88 (each 1H, br s, 8-OH and 9-OH), 4.19 (1H, br s, 6-OH), 4.11 (1H, br s, 10-OH), 3.66 (1H, m, H-6), 3.65 (1H, d, *J* = 11.2 Hz, H-8), 3.49 (1H, br d, *J* = 11.2 Hz, H-9), 2.25 (1H, m, H-11), 1.84 (1H, d, *J* = 6.0 Hz, H-1), 1.79 (1H, m, H-3), 1.72 (1H, d, *J* = 6.8 Hz, H-2), 1.67 (1H, m, H-4β), 1.57 (1H, m, H-5β), 1.53 (1H, m, H-12β), 1.52 (1H, m, H-5α), 1.50 (1H, m, H-4α), 1.49 (1H, m, H-12α), 1.48 (3H, s, H_3_-16), 1.41 (1H, m, H-13β), 1.37 (^1^H, m, H-13α), 1.365 (3H, s, H_3_-17), 1.16 (3H, d, *J* = 6.8 Hz, H_3_-20), 1.06 (3H, s, H_3_-19), 1.03 (3H, d, *J* = 6.8 Hz, H_3_-18). ESIMS *m/z* 377 [M + Na]^+^; HRESIMS *m/z* 377.2300 [M + Na]^+^ (calcd for C_20_H_34_O_5_Na, *m/z* 377.2299).

Briarol B (2). White powder; ${\left[\alpha \right]}_{D}^{25}$ −32.0 (c 0.15, CHCl_3_); IR (neat) ν_max_ 3464, 2923, 2854, and 1381 cm^−1^; ^13^C NMR (125 MHz, CDCl_3_) and ^1^H NMR (500 MHz, CDCl_3_). See Table 1 and Table 2, respectively. ESIMS *m/z* 361 [M + Na]^+^, 339 [M + H]^+^ HRESIMS *m/z* 361.2348 [M + Na]^+^ (calcd for C_20_H_34_O_4_Na, *m/z* 361.2349).

Briarol C (3). White powder; ${\left[\alpha \right]}_{D}^{25}$ −21.3 (c 0.22, CHCl_3_); IR (neat) ν_max_ 3413, 2925, 2858, and 1374 cm^−1^; ^13^C NMR (100 MHz, CDCl_3_) and ^1^H NMR (500 MHz, CDCl_3_). See Table 1 and Table 2, respectively. ESIMS *m/z* 361 [M + Na]^+^; HRESIMS *m/z* 361.2347 [M + Na]^+^ (calcd for C_20_H_34_O_4_Na, *m/z* 361.2349).

Briarol D (4). White powder; ${\left[\alpha \right]}_{D}^{25}$ −29.7 (c 0.22, CHCl_3_); IR (neat) ν_max_ 3418, 2923, 2853, and 1381 cm^−1^; ^13^C NMR (150 MHz, acetone-*d_6_*) and ^1^H NMR (600 MHz, acetone-*d_6_*), see Table 1 and Table 2, respectively. ESIMS *m/z* 361 [M + Na]^+^; HRESIMS *m/z* 361.2349 [M + Na]^+^ (calcd for C_20_H_34_O_4_Na, *m/z* 361.2349).

### 3.4. Cytotoxicity Assay

Cancer cell lines (HT-29, HuCC-T1, and DLD-1) were obtained from the American Type Culture Collection (ATCC). Compounds 1‒4 were evaluated for the cytotoxic activity using an Alamar blue assay as previously described [28,29]. The intensity of the produced color was measured at 570 nm using an ELISA plate reader.

## 4. Conclusions

Three new polyoxygenated diterpenoids of the rare klysimplexane-skeleton, along with a non-ether bridged eunicellin diterpenoid, were discovered from the gorgonian coral *Briareum violaceum* and named briarols A‒D, respectively. A possible biosynthetic pathway for briarols A‒C from the coexisting eunicellin diterpenoid was postulated for the first time. Although the compounds did not show potent cytotoxic activity against the tested cancer lines, other possible bioactivities for these metabolites might be worthwhile for further screening. It is noteworthy to mention that this is the first discovery of these rare klysimplexane-type metabolites from a gorgonian coral since the isolation of klysimplexin T from the cultured soft coral *Klyxum simplex* a decade ago.

## Figures and Tables

**Table 1:** ^13^C NMR spectroscopic data of compounds 1–4.

#	1 ^a^	2 ^b^	3 ^b^	4 ^c^
1	43.5 (CH)^d)^	41.6 (CH)	45.2 (CH)	50.1 (CH)
2	44.3 (CH)	45.6 (CH)	42.3 (CH)	125.5 (CH)
3	28.5 (CH)	144.1 (C)	27.9 (CH)	137.7 (C)
4	32.3 (CH_2_)	36.6 (CH_2_)	31.2 (CH_2_)	38.1 (CH_2_)
5	25.4 (CH_2_)	33.3 (CH_2_)	24.8 (CH_2_)	39.0 (CH_2_)
6	81.9 (CH)	81.5 (CH)	81.4 (CH)	76.2 (CH)
7	44.5 (C)	46.4 (C)	43.8 (CH)	162.8 (C)
8	80.2 (CH)	78.4 (CH)	79.8 (CH)	70.7 (CH)
9	79.8 (CH)	78.2 (CH)	78.8 (CH)	80.9 (CH)
10	78.9 (C)	76.0 (C)	77.2 (C)	78.3 (C)
11	32.9 (CH)	32.1 (CH)	32.3 (CH)	33.2 (CH)
12	30.6 (CH_2_)	33.6 (CH_2_)	33.8 (CH_2_)	33.8 (CH_2_)
13	25.8 (CH_2_)	22.0 (CH_2_)	25.3 (CH_2_)	26.1 (CH_2_)
14	70.9 (C)	43.9 (CH)	128.3 (C)	44.4 (CH)
15	64.2 (C)	28.5 (CH)	128.0 (C)	31.4 (CH)
16	23.6 (CH_3_)	23.3 (CH_3_)	20.9 (CH_3_)	22.0 (CH_3_)
17	20.8 (CH_3_)	21.9 (CH_3_)	20.4 (CH_3_)	22.5 (CH_3_)
18	17.2 (CH_3_)	111.8 (CH_2_)	15.2 (CH_3_)	18.3 (CH_3_)
19	10.9 (CH_3_)	9.2 (CH_3_)	9.9 (CH_3_)	113.8 (CH_2_)
20	17.9 (CH_3_)	17.2 (CH_3_)	17.1 (CH_3_)	17.8 (CH_3_)

Spectrum recorded at ^a^ 100 MHz in C_6_D_6_, ^b^ 125 MHz in CDCl_3_, and ^c^ 150 MHz in acetone-*d_6_*. ^d^ Attached protons were deduced by distortionless enhancement by polarization transfer (DEPT) and heteronuclear single quantum correlation (HSQC) experiments.

**Table 2:** ^1^H NMR spectroscopic data of compounds 1–4.

No	1 ^a^	2 ^b^	3 ^b^	4 ^c^
1	1.64 d (12.0) ^d^	2.08 m	2.81 d(7.5)	2.52 dd (10.0, 3.0)
2	1.23 dd (12.0, 3.6)	2.32 d (12.5)	1.62 m	5.35 d (10.0)
3	1.40 m	-	1.54 m	-
4α	1.21 m	2.23 dd (7.5, 5.0)	1.52 m	2.15 m
4β	1.28 m	2.00 m	1.52 m	2.26 m
5α	1.80 m	1.93 dd (12.0, 5.0)	1.61–1.64 m	1.91 m
5β	1.80 m	2.34 m	1.61-1.64 m	2.04 m
6	3.54 dd (8.0, 8.0)	3.80 dd (11.5, 5.0)	3.61 dd (11.0,4.5)	4.08 d (7.5)
8	3.44 d (11.2)	3.84 d (11.0)	3.64 d (11.0)	4.21 d (4.5)
9	3.49 d (11.2)	3.56 d (11.0)	3.66 d (11.0)	3.46 d (5.5)
11	1.79 m	1.99 m	2.14 m	1.89 m
12α	1.79 m	1.32 m	1.55 m	1.41 m
12β	1.81 m	1.54 m	1.14 m	1.46 m
13α	1.06 m	1.56 m	2.47 m	1.59 m
13β	1.22 m	1.56 m	1.64 m	1.59 m
14	-	1.73 m	-	1.54 m
15	-	2.11 m	-	1.25 m
16	1.08 3H, s	0.92 3H, d (6.5)	1.81 3H, s	0.82 3H, d (6.0)
17	0.94 3H, s	0.73 3H, d (6.5)	1.74 3H, s	0.81 3H, d (6.0)
18	0.68 3H, d (7.2)	5.02 s 4.81 s	0.80 3H, d (7.5)	1.57 3H, s
19	1.09 3H, s	0.93 3H, s	1.11 3H, s	5.43 d (1.2) 5.37 d (1.2)
20	1.28 3H, d (5.6)	1.04 3H, d (6.5)	1.07 3H, d (6.0)	0.92 3H, d (6.6)
6-OH	4.12, br s		-	3.62 br s
8-OH	2.94, br s		-	4.26 br d (4.5)
9-OH	2.32, br s		2.91 br s*	4.00 d (5.5)
10-OH	4.54, br s		3.18 br s*	3.57, s

Spectrum recorded at ^a^ 400 MHz in C_6_D_6_, ^b^ 500 MHz in CDCl_3_, and ^c^ 600 MHz in acetone-*d_6_*. ^d^ *J* values (Hz). * Exchangeable data.

## Data Availability

Data available in a publicly accessible repository.

## Supplementary Materials

Figure S1. HRESIMS spectrum of 1; Figures S2–S7: 1D and 2D NMR spectra of 1 in C_6_D_6_; Figures S8–S13. 1D and 2D NMR spectra of 1 in CDCl_3_; Figures S14–S19. 1D and 2D NMR spectra of 1 in acetone-*d_6_*; Figure S20. HRESIMS spectrum of 2; Figures S21–S26. 1D and 2D NMR spectra of 2 in CDCl_3_; Figure S27. HRESIMS spectrum of 3; Figures S28–S33. 1D and 2D NMR spectra of 3 in CDCl_3_; Figure S34. HRESIMS spectrum of 4; Figures S35–S40. 1D and 2D NMR spectra of 4 in in acetone-*d_6._*

## Sample Availability

Samples are not available from the authors as they were consumed during measurements and bioassays.
