# Supplementary material for: Polyoxygenated Klysimplexane- and Eunicellin-Based Diterpenoids from the Gorgonian Briareum violaceum
Source: Molecules. 2021 May 28;26(11):3276. doi: 10.3390/molecules26113276 (PMC8198191; doi:10.3390/molecules26113276)
Supplement: Supplementary file 1 [file molecules-26-03276-s001.zip › molecules-1164366-supplementary.pdf]

molecules

MDPI

Supplementary Materials

Polyoxygenated Klysimplexane- and Eunicellin-Based Diterpenoids from the Gorgonian *Briareum violaceum*

Atallah F. Ahmed [1,2,†], Yang Cheng [3,†], Chang-Feng Dai [4] and Jyh-Horng Sheu [3,5,6,7,*]

[1] Department of Pharmacognosy, College of Pharmacy, King Saud University, Riyadh 11451, Saudi Arabia; afahmed@ksu.edu.sa
[2] Department of Pharmacognosy, Faculty of Pharmacy, Mansoura University, Mansoura 35516, Egypt; afahmed@ksu.edu.sa
[3] Department of Marine Biotechnology and Resources, National Sun Yat-sen University, Kaohsiung 804, Taiwan; jack1991106@yahoo.com.tw (Y.C.); sheu@mail.nsysu.edu.tw (J. -H. S.)
[4] Institute of Oceanography, National Taiwan University, Taipei 112, Taiwan; corallab@ntu.edu.tw
[5] Graduate Institute of Natural Products, Kaohsiung Medical University, Kaohsiung 807, Taiwan; sheu@mail.nsysu.edu.tw
[6] Frontier Center for Ocean Science and Technology, National Sun Yat-sen University, Kaohsiung 804, Taiwan; sheu@mail.nsysu.edu.tw
[7] Department of Medical Research, China Medical University Hospital, China Medical University, Taichung 404, Taiwan; sheu@mail.nsysu.edu.tw
* Correspondence: Correspondence: sheu@mail.nsysu.edu.tw; Tel.: +886-7-525-2000 (ext. 5030); Fax: +886-7-525-5020
† These authors contributed equally to this work.

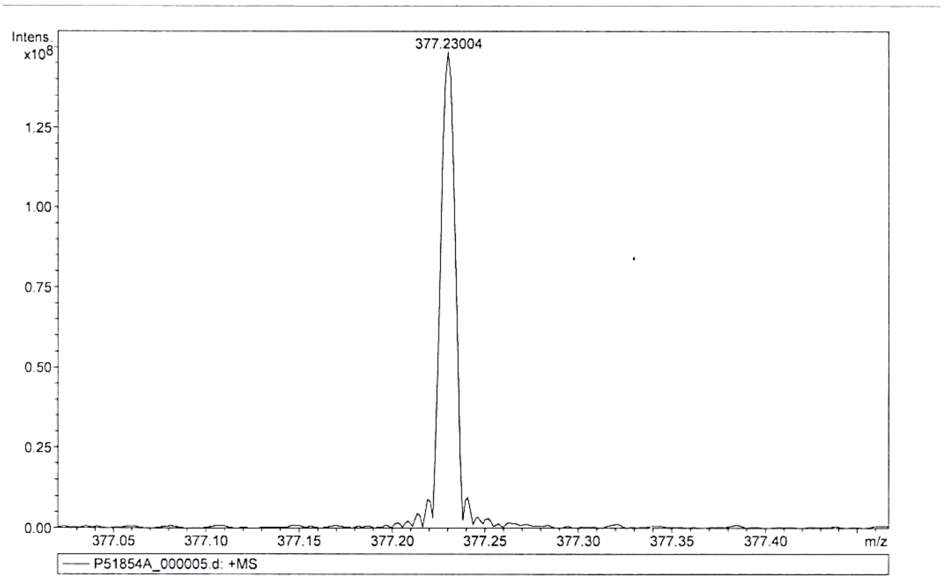

Meas. m/z	#	Formula	Score	m/z	err [mDa]	err [ppm]	mSigma	rdb	e⁻ Conf	N-Rule
377.23004	1	C 20 H 34 Na O 5	100.00	377.22985	-0.19	-0.51	3.7	3.5	even	ok

Figure S1. HRESIMS spectrum of **1**.

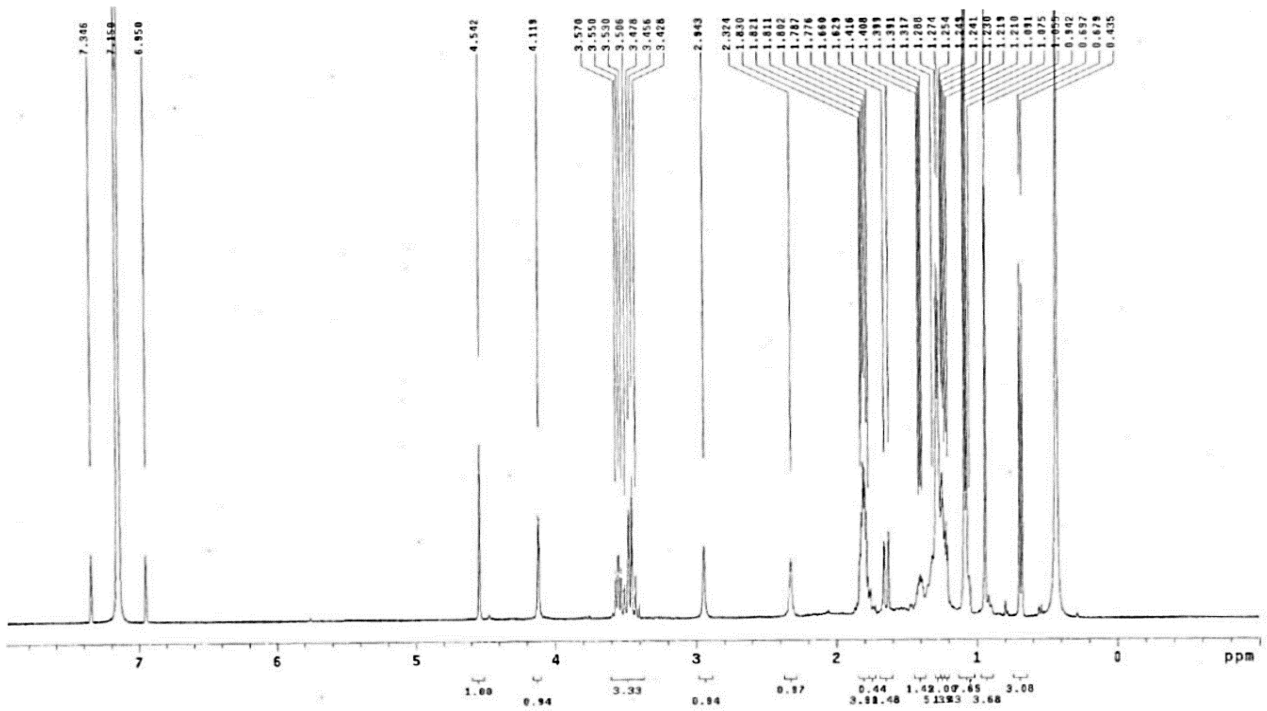

Figure S2. ^{1}H NMR spectrum of **1** in C_6D_6 at 400 MHz.

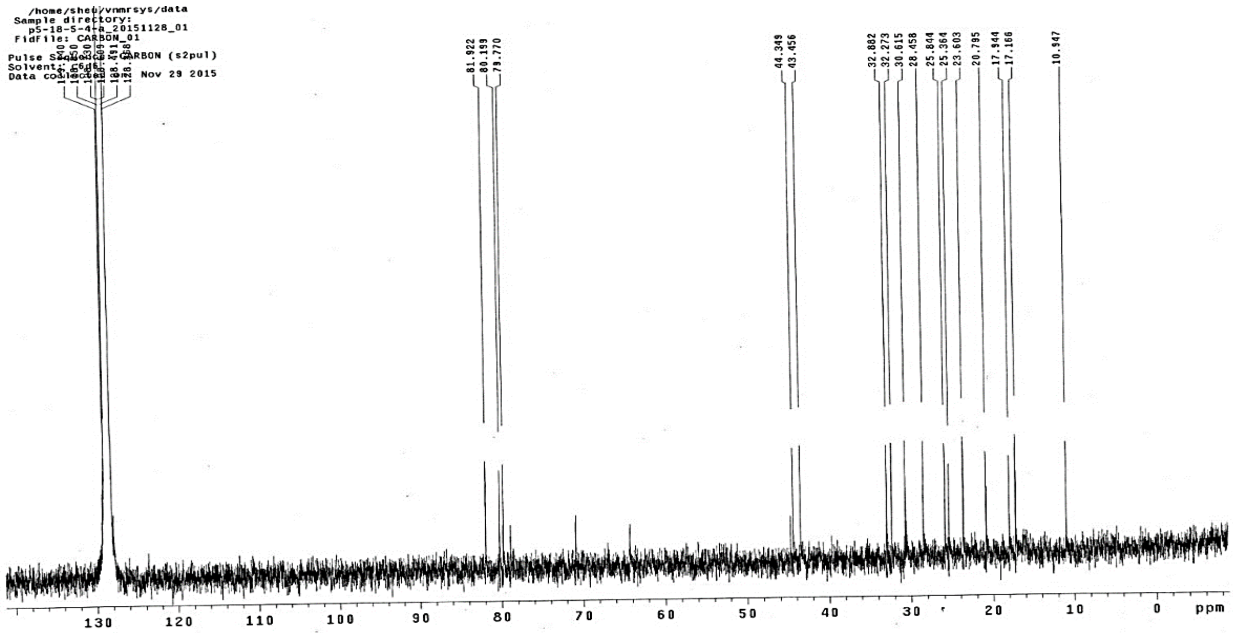

Figure S3. ^{13}C NMR spectrum of **1** in C_6D_6 at 100 MHz.

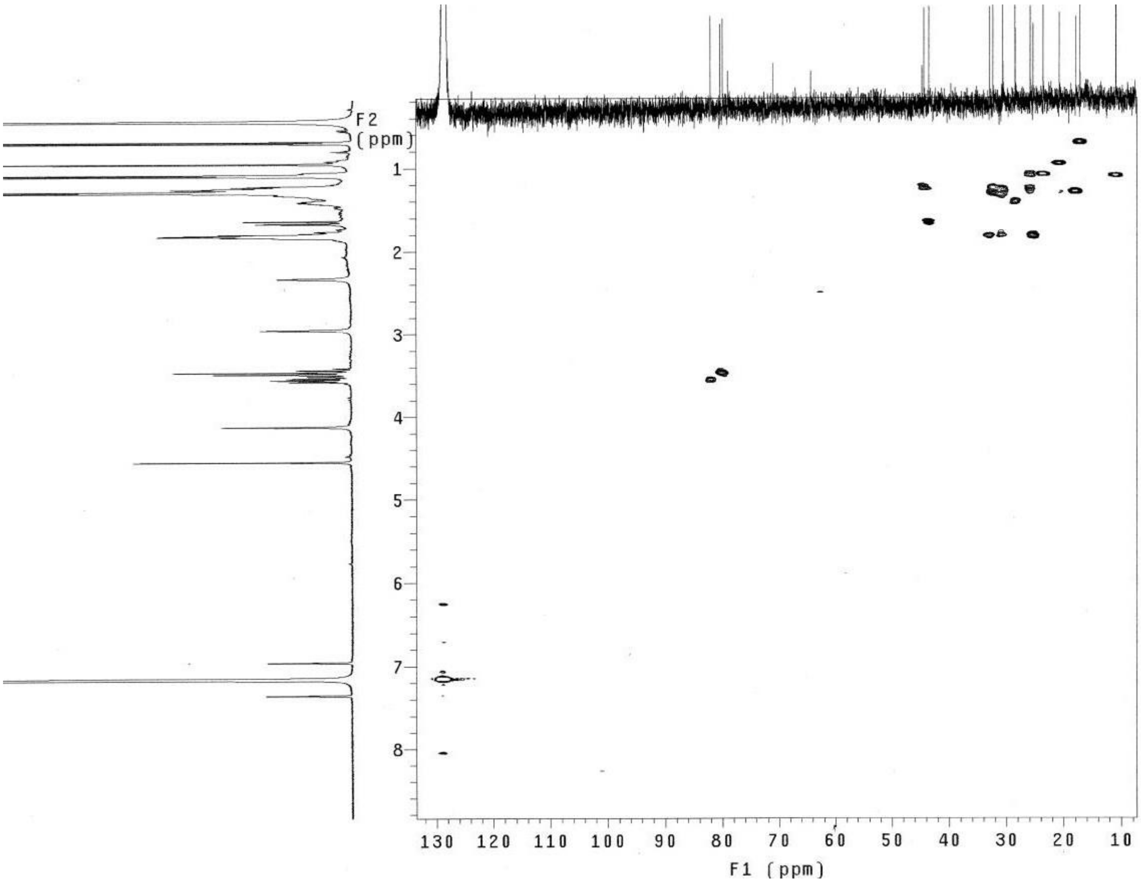

Figure S4. HMQC spectrum of **1** in C_6D_6.

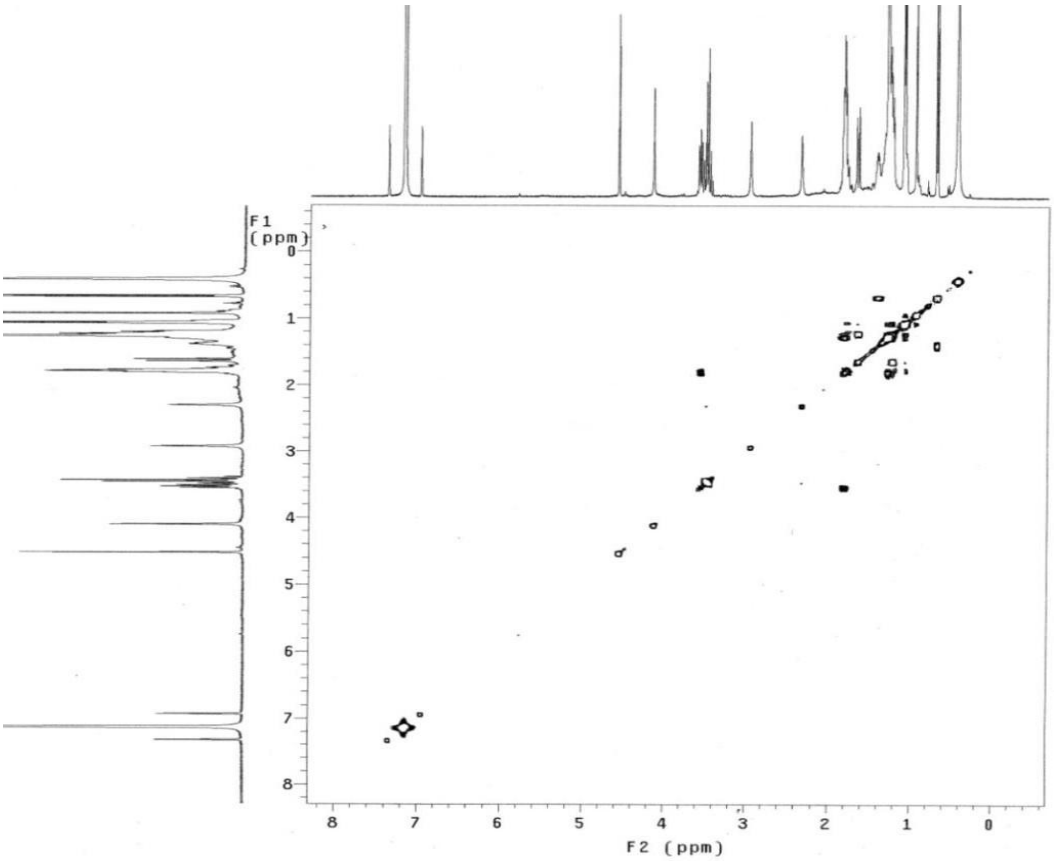

Figure S5. COSY spectrum of **1** in C_6D_6.

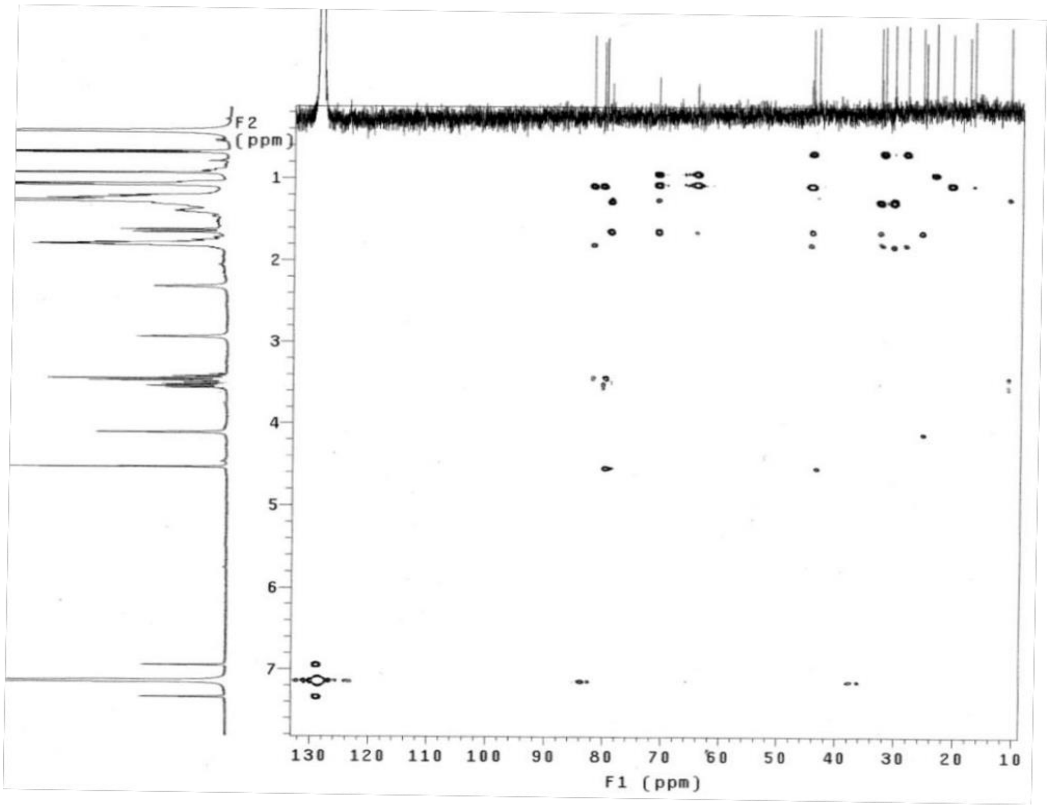

Figure S6. HMBC spectrum of **1** in C_6D_6.

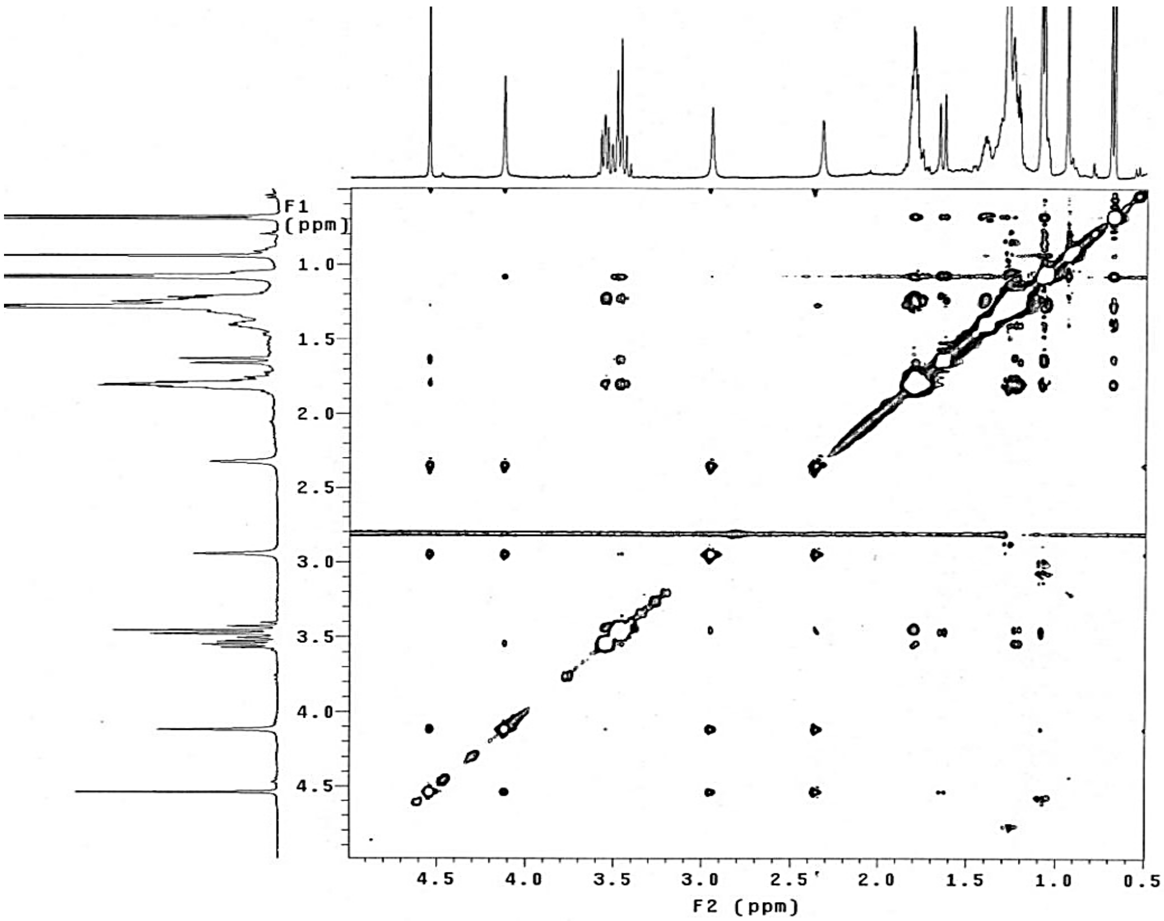

Figure S7. NOESY spectrum of **1** in C_6D_6.

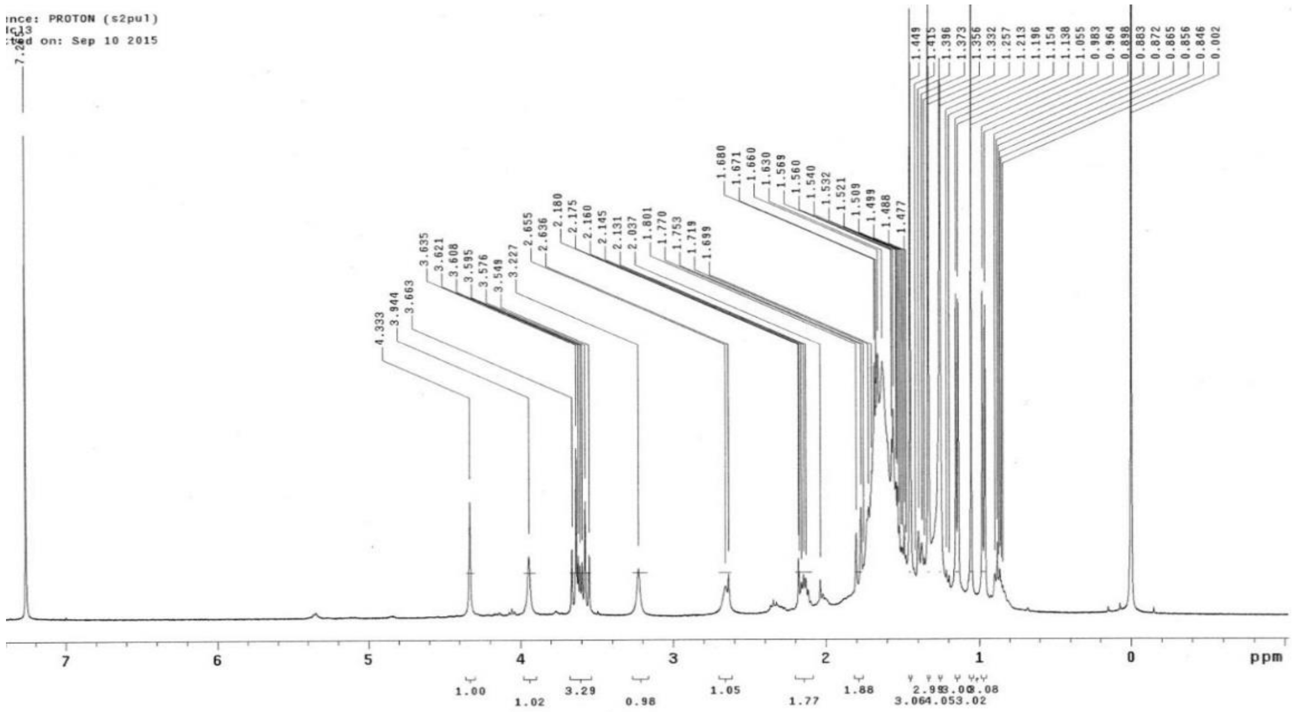

Figure S8. ^{1}H NMR spectrum of **1** in $CDCl_3$ at 400 MHz.

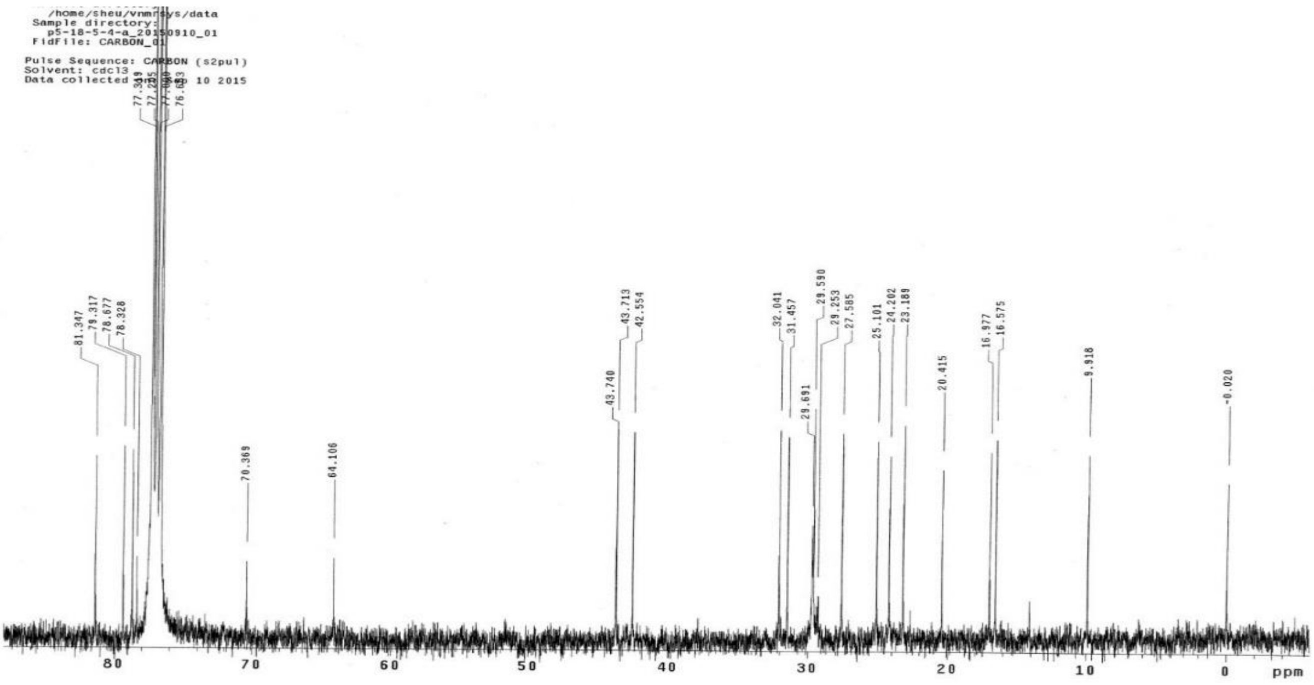

Figure S9. ^{13}C NMR spectrum of **1** in $CDCl_3$ at 100 MHz.

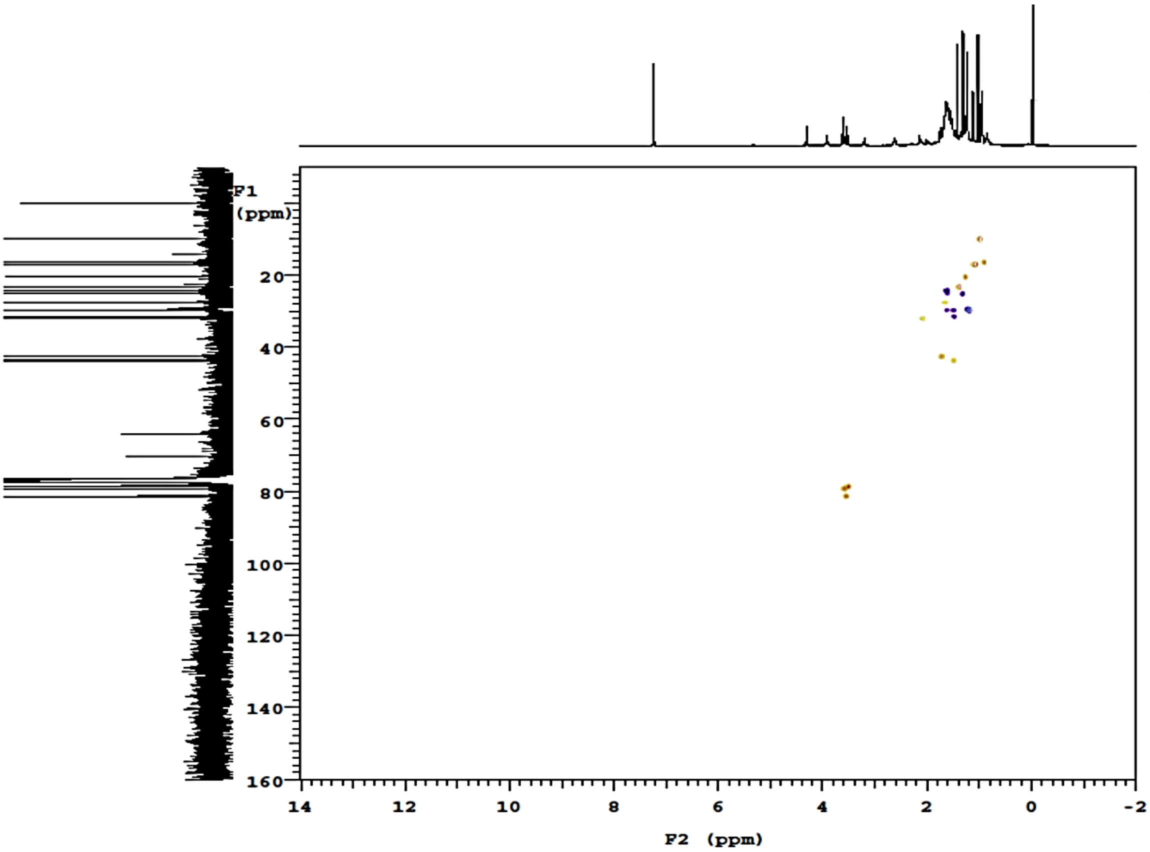

Figure S10. HMQC spectrum of **1** in $CDCl_3$.

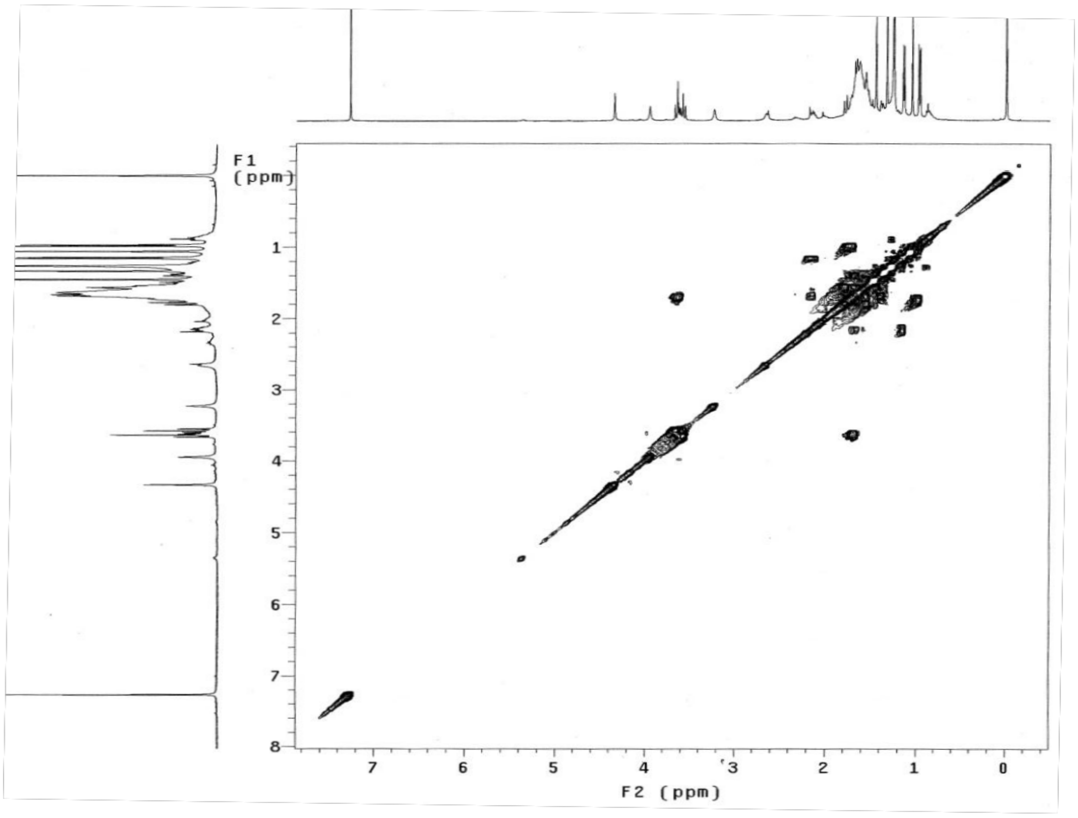

Figure S11. COSY spectrum of **1** in CD Cl_3.

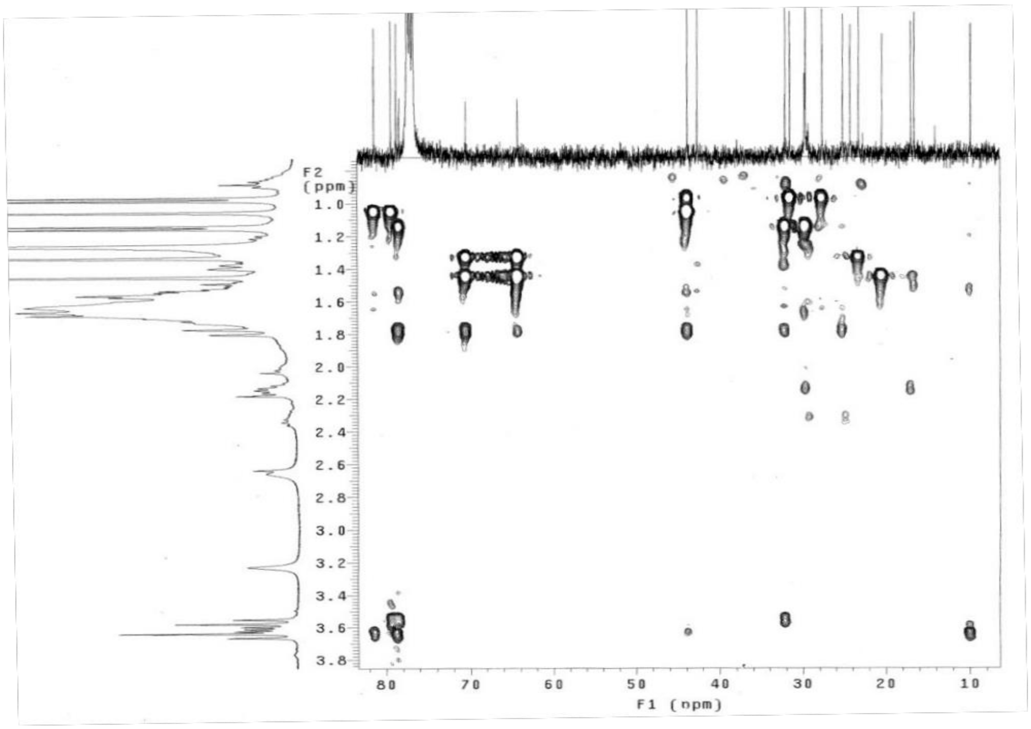

Figure S12. HMBC spectrum of **1** in $CDCl_3$.

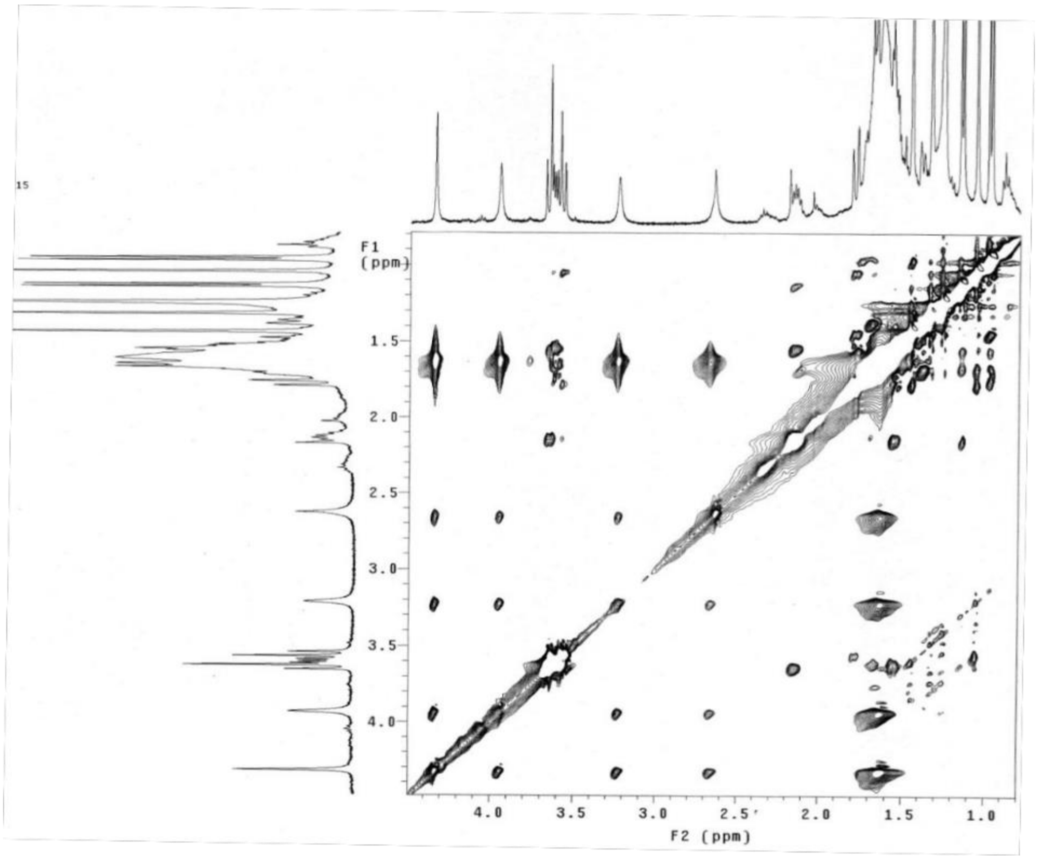

Figure S13. NOESY spectrum of **1** in $CDCl_3$.

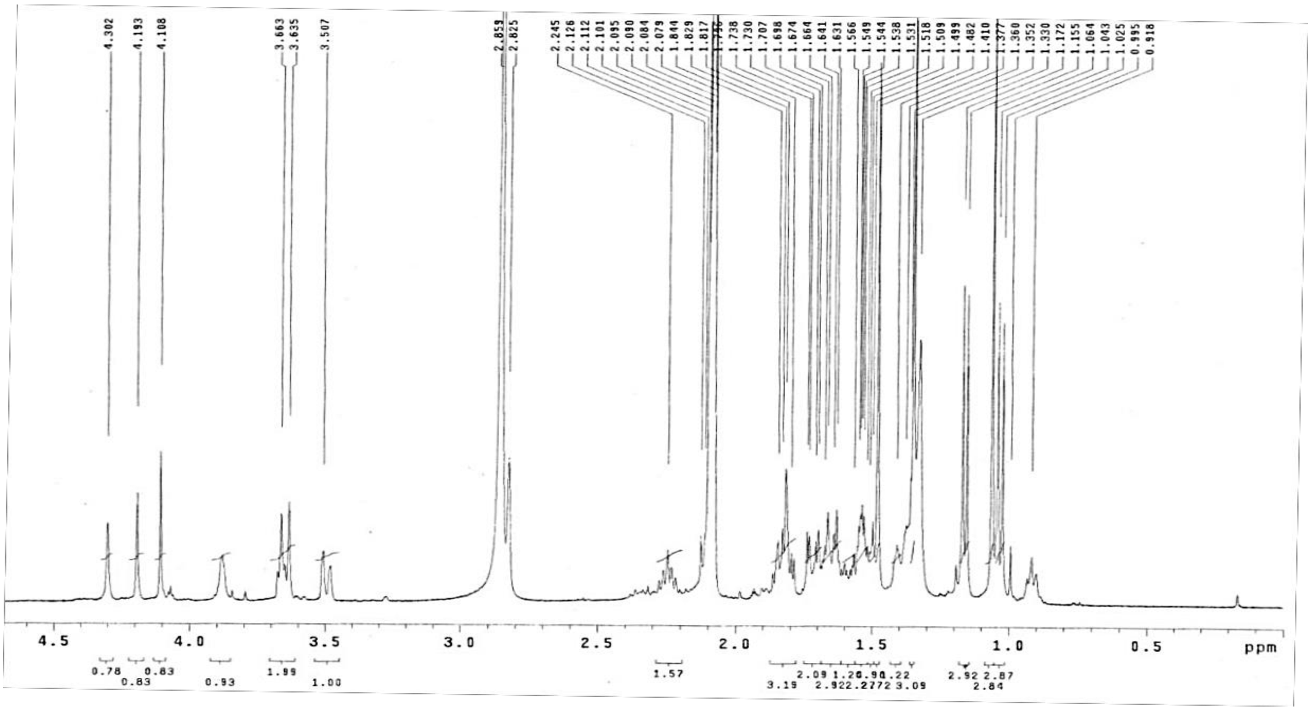

Figure S14. ^1H NMR spectrum of **1** in acetone-d_6 at 400 MHz.

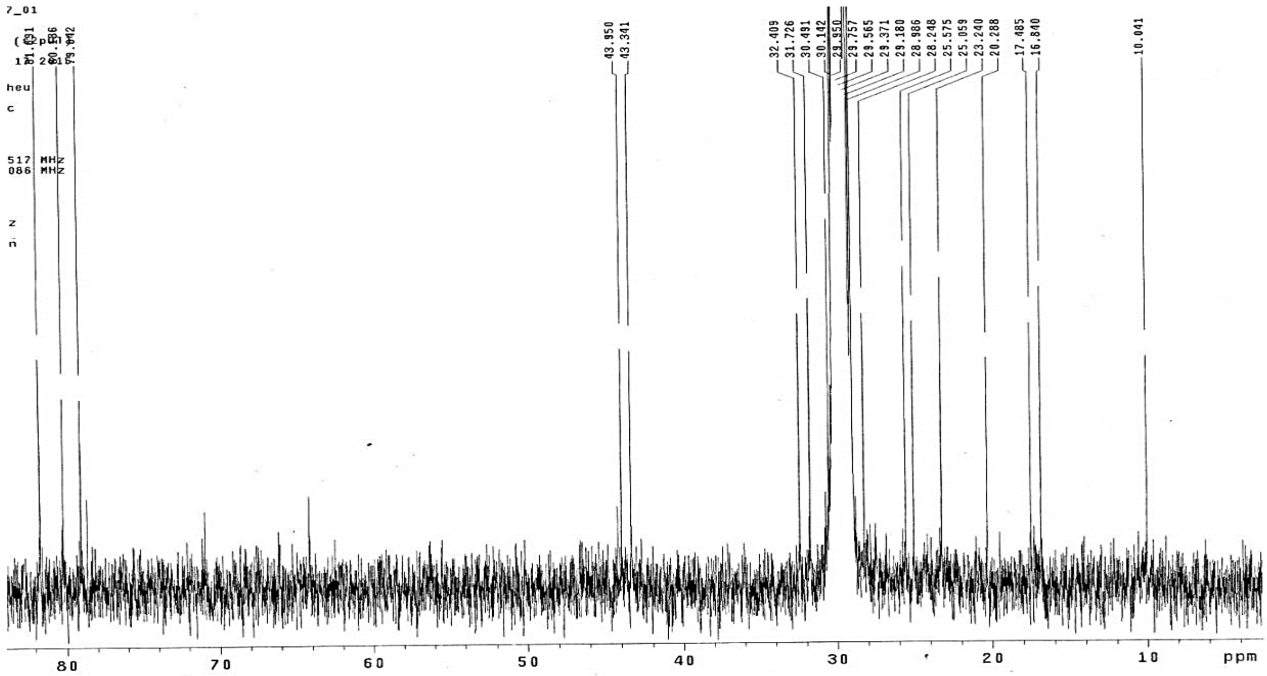

Figure S15. ^{13}C NMR spectrum of **1** in acetone-d_6 at 100 MHz.

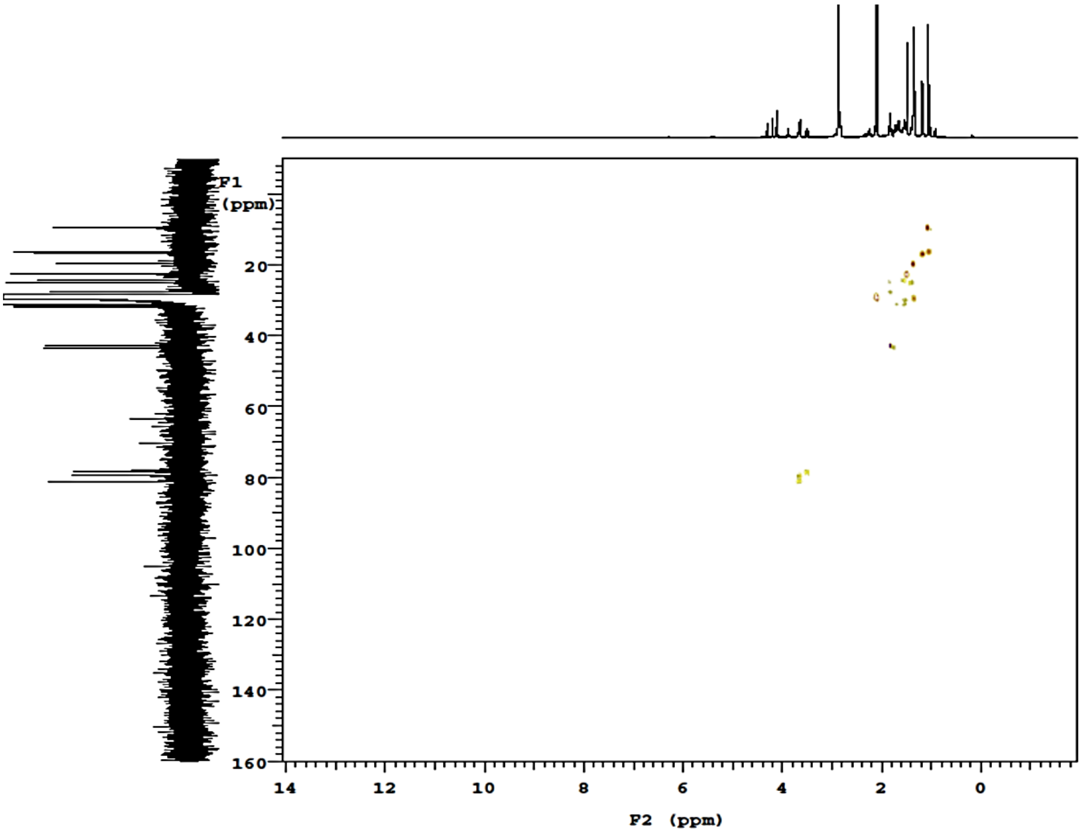

Figure S16. HMQC spectrum of **1** in acetone-d_6.

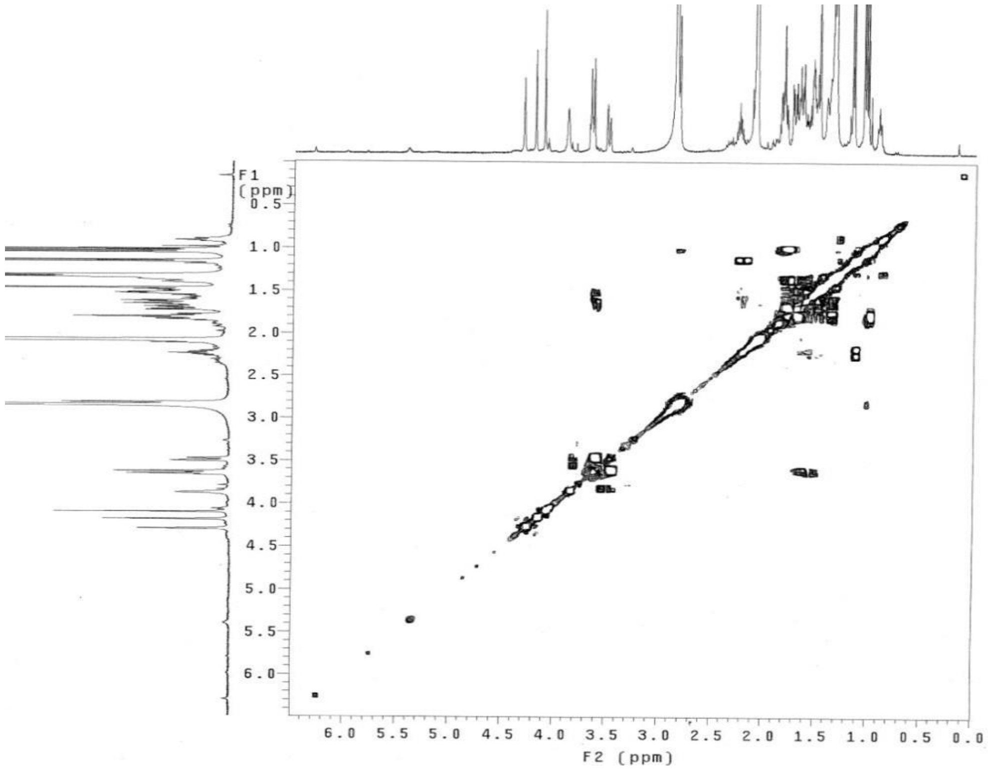

Figure S17. COSY spectrum of **1** in acetone-d_6.

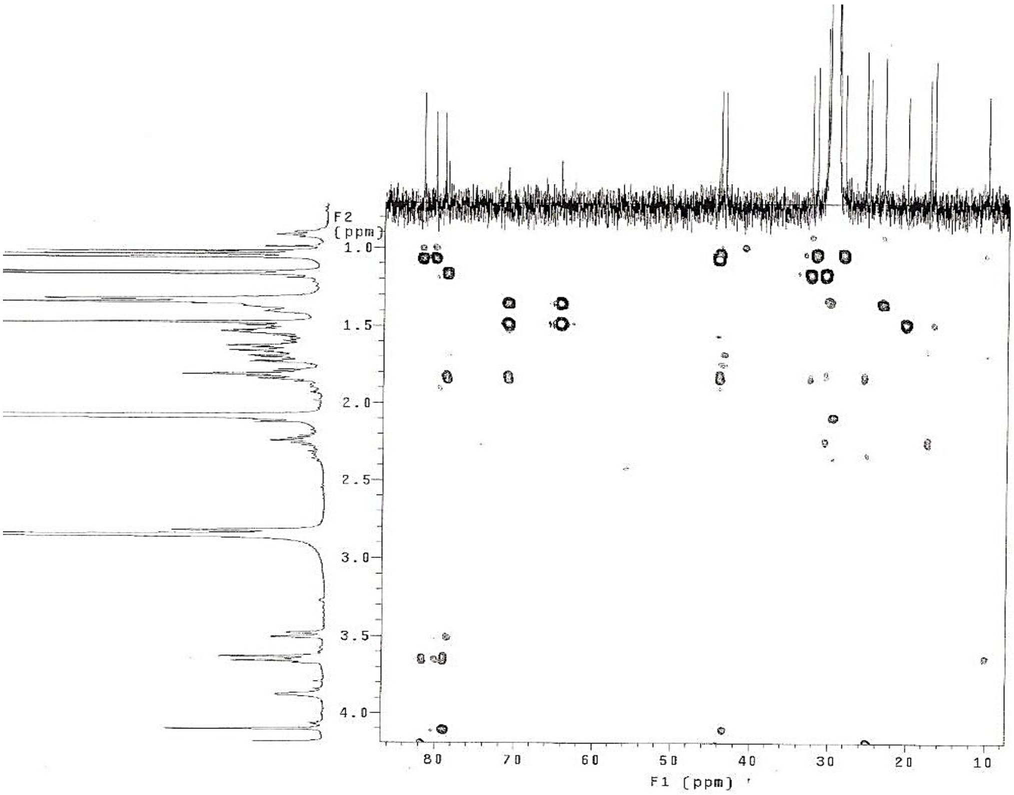

Figure S18. HMBC spectrum of **1** in acetone-d_6.

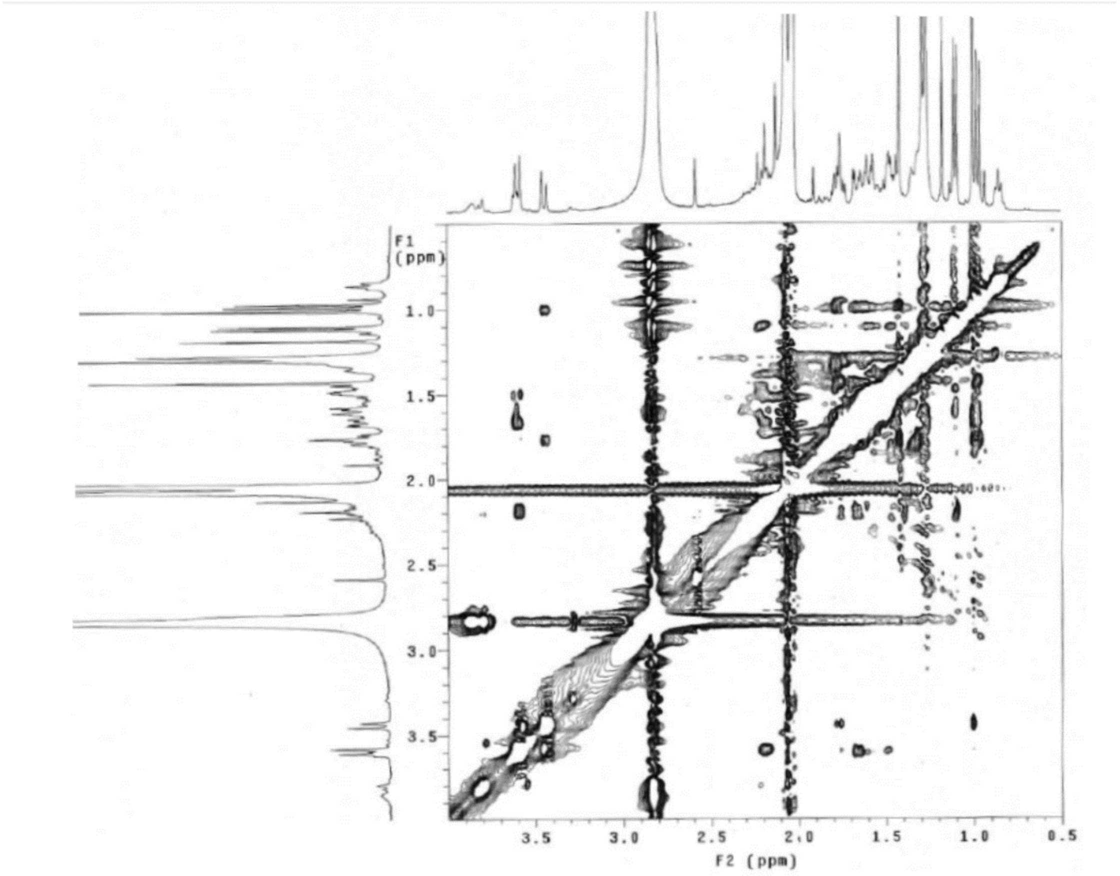

Figure S19. NOESY spectrum of **1** in acetone-d_6.

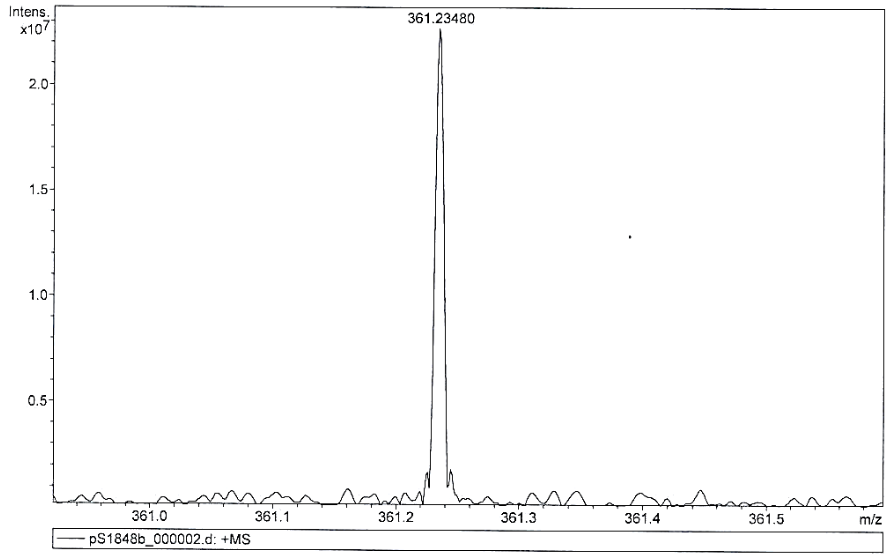

Meas. m/z	#	Formula	Score	m/z	err [mDa]	err [ppm]	mSigma	rdb	e⁻ Conf	N-Rule
361.23480	1	C 20 H 34 Na O 4	100.00	361.23493	0.13	0.37	20.3	3.5	even	ok

Figure S20. HRESIMS spectrum of **2**.

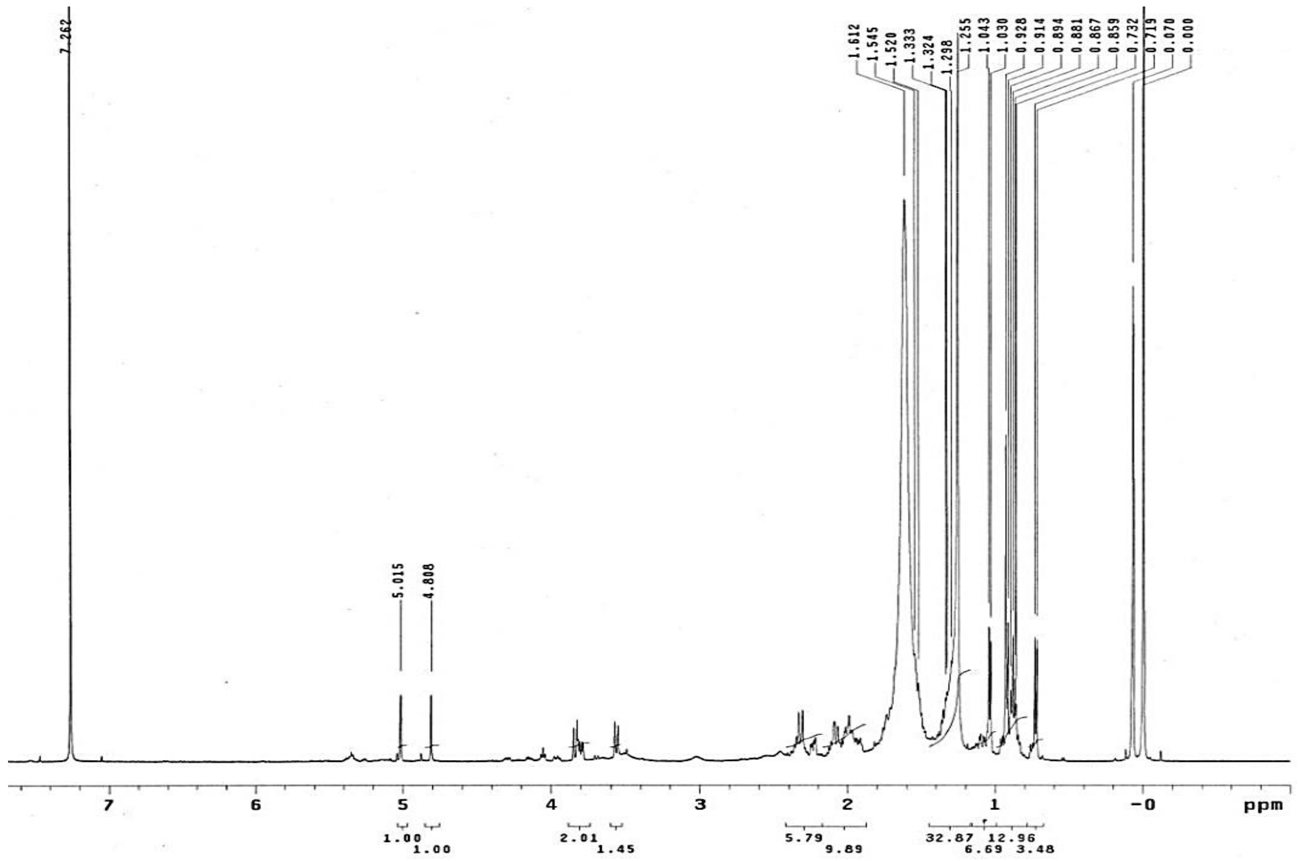

Figure S21. ^1H NMR spectrum of **2** in $CDCl_3$ at 500 MHz.

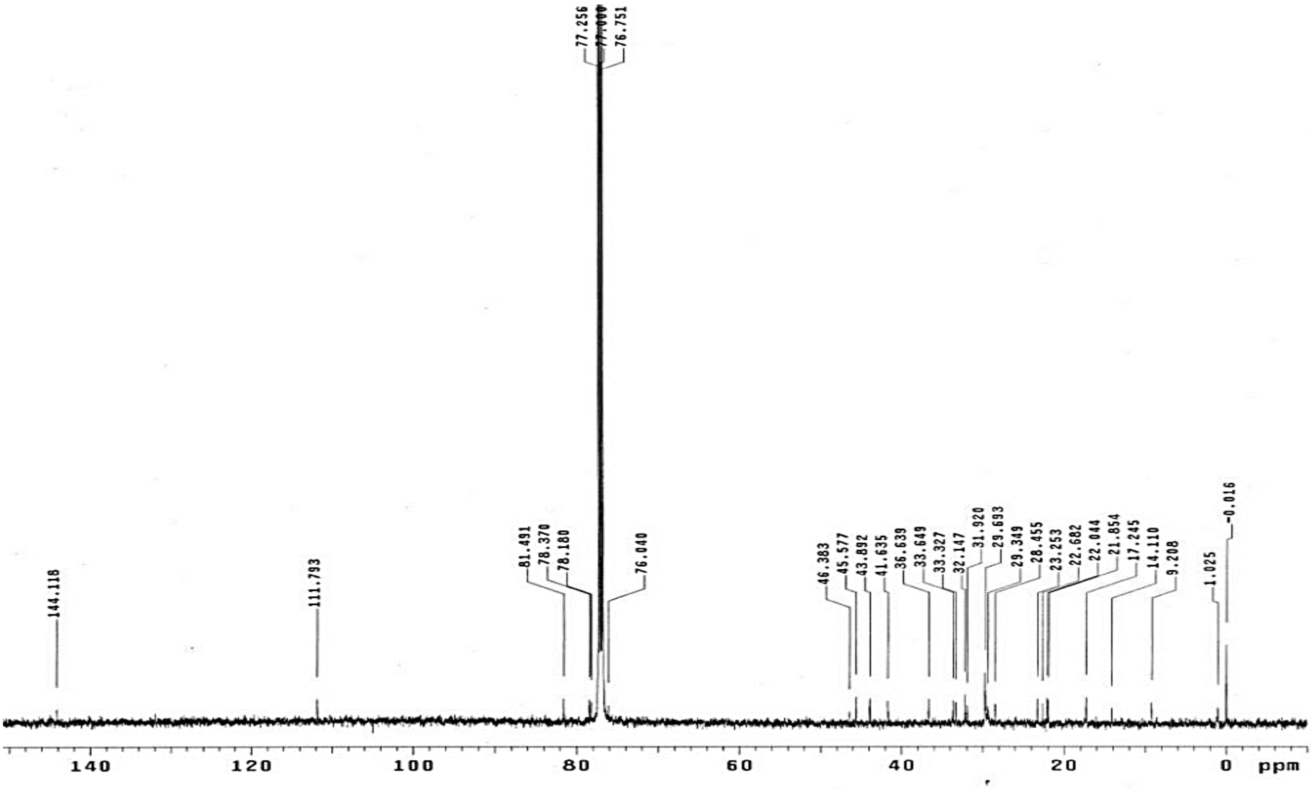

Figure S22. ^{13}C NMR spectrum of **2** in $CDCl_3$ at 125 MHz.

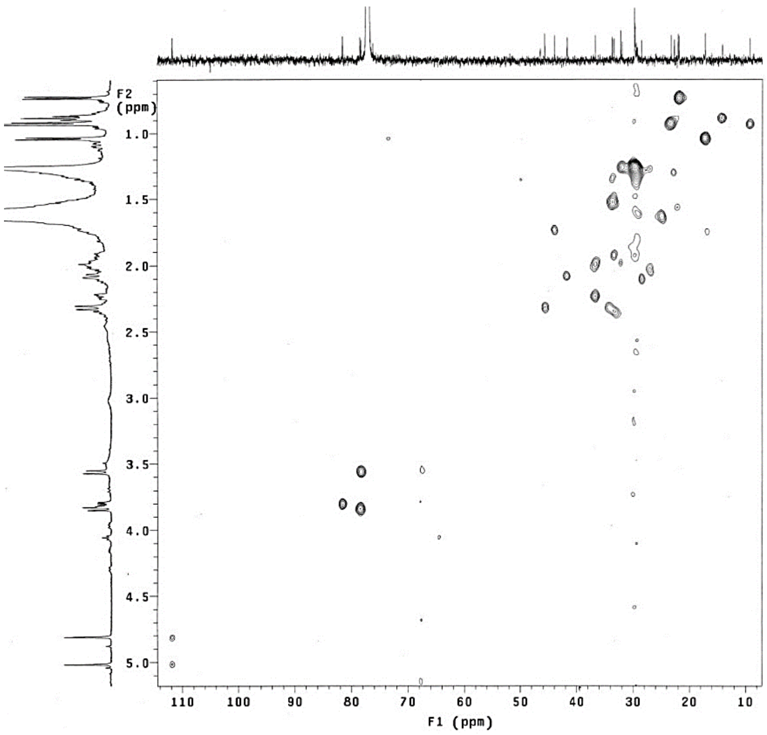

Figure S23. HSQC spectrum of **2** in $CDCl_3$.

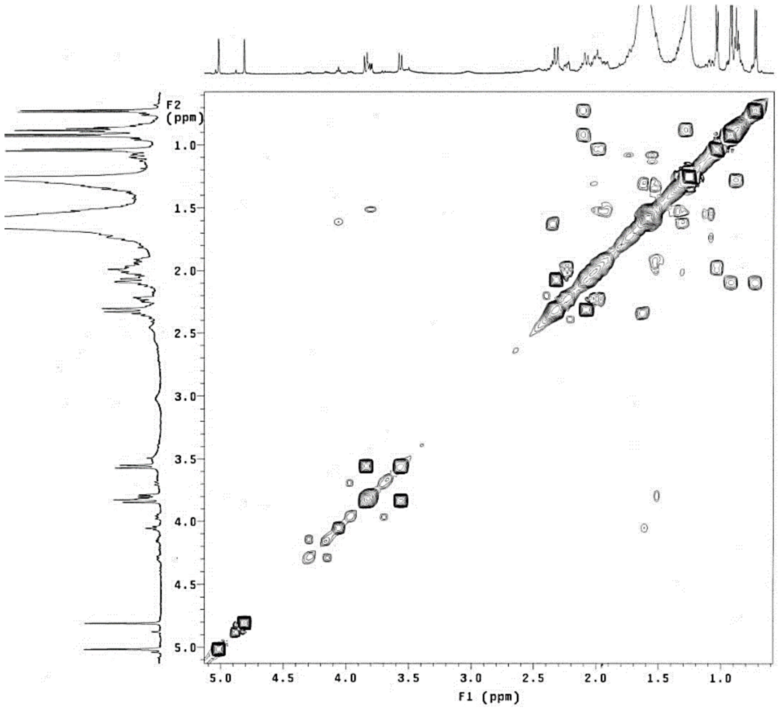

Figure S24. COSY spectrum of **2** in $CDCl_3$.

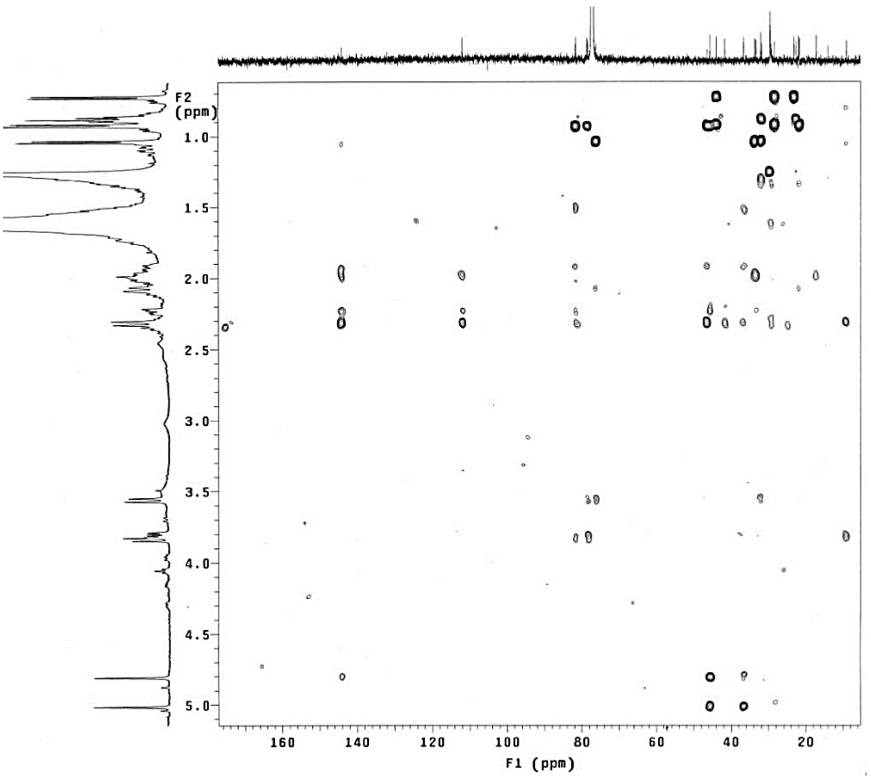

Figure S25. HMBC spectrum of **2** in $CDCl_3$.

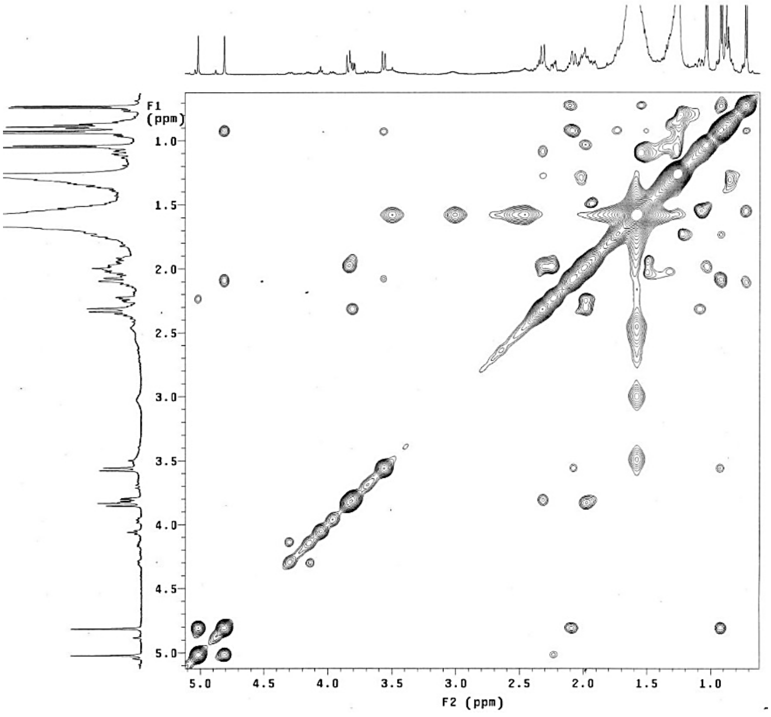

Figure S26. NOESY spectrum of **2** in $CDCl_3$.

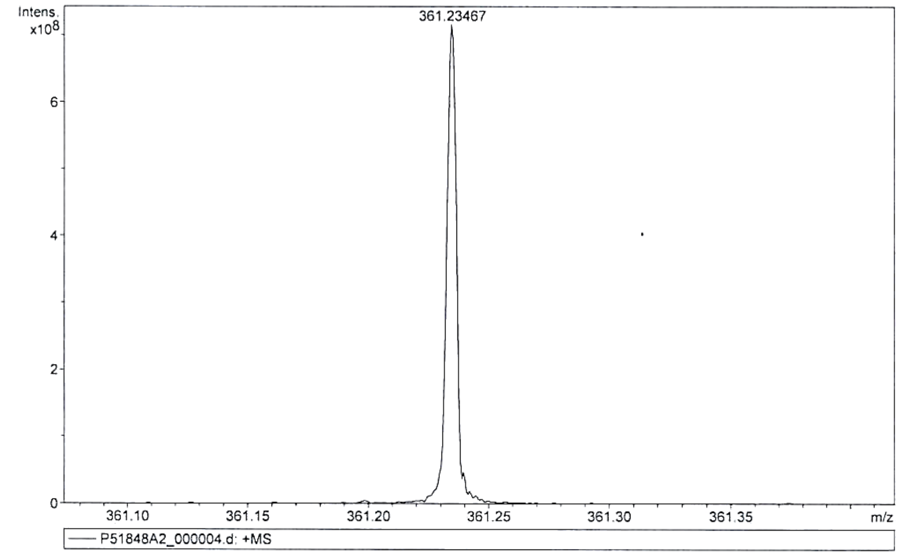

Meas. m/z	#	Formula	Score	m/z	err [mDa]	err [ppm]	mSigma	rdb	e⁻ Conf	N-Rule
361.23467	1	C 20 H 34 Na O 4	100.00	361.23493	0.26	0.73	9.9	3.5	even	ok

Figure S27. HRESIMS spectrum of **3.**

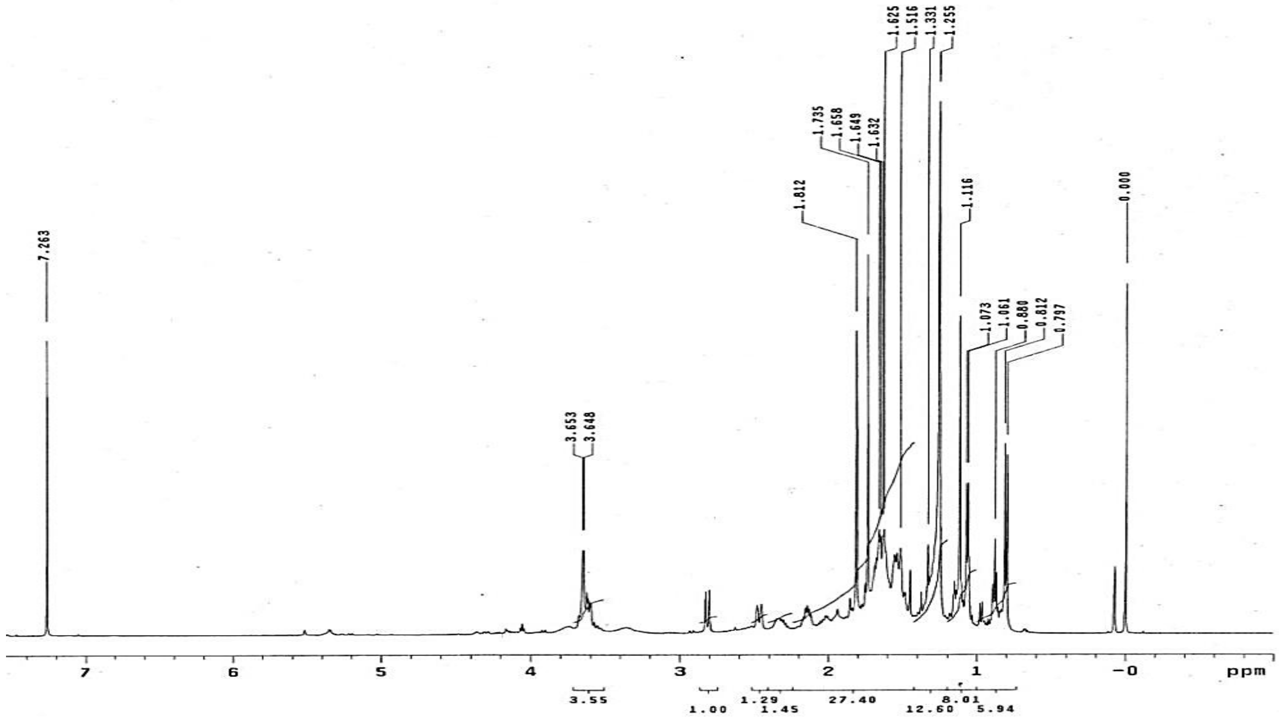

Figure S28. ^{1}H NMR spectrum of **3** in $CDCl_3$ at 500 MHz.

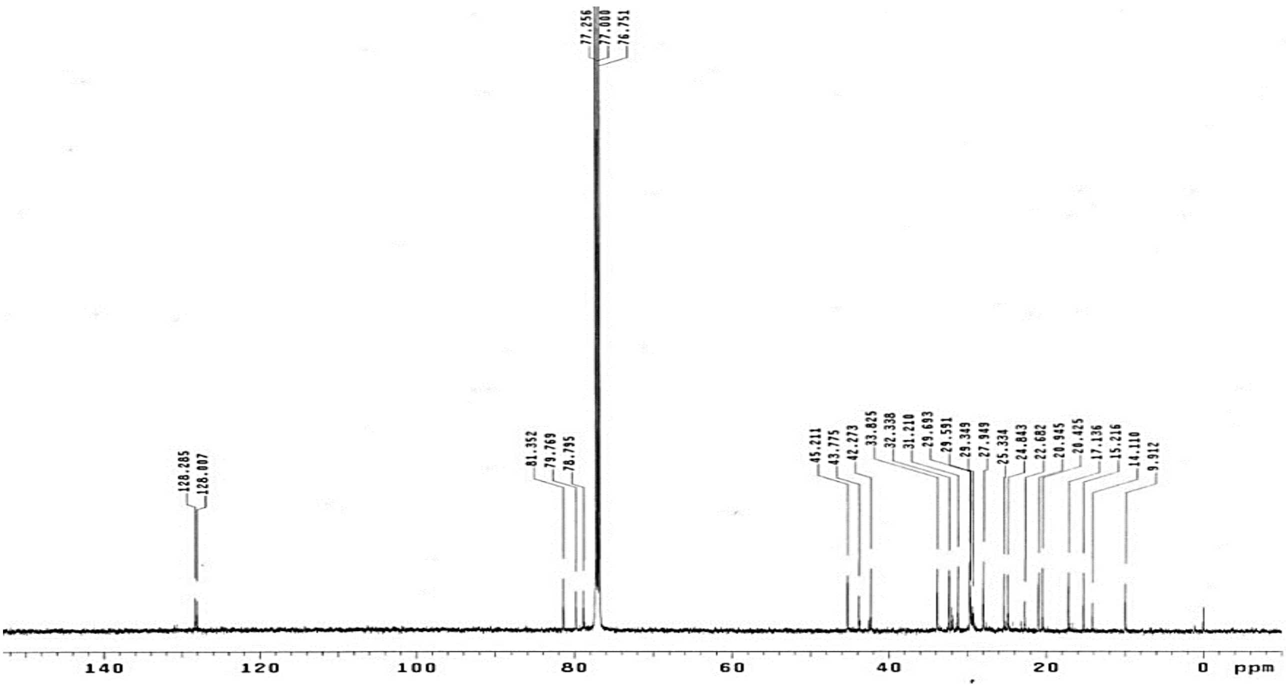

Figure S29. ^{13}C NMR spectrum of **3** in $CDCl_3$ at 125 MHz.

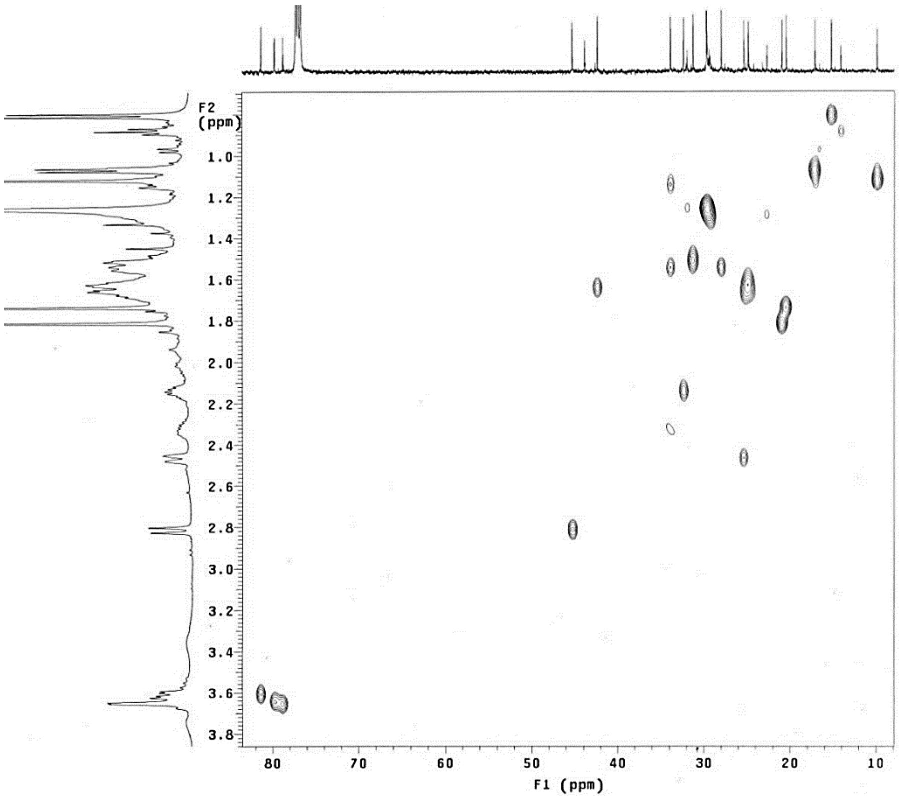

Figure S30. HSQC spectrum of **3** in $CDCl_3$.

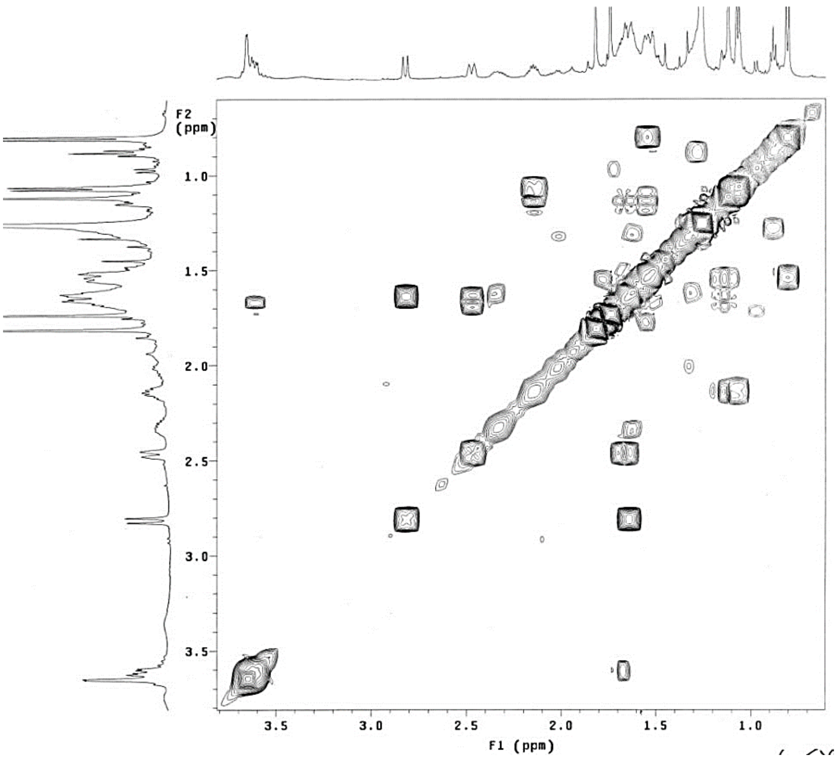

Figure S31. COSY spectrum of **3** in $CDCl_3$.

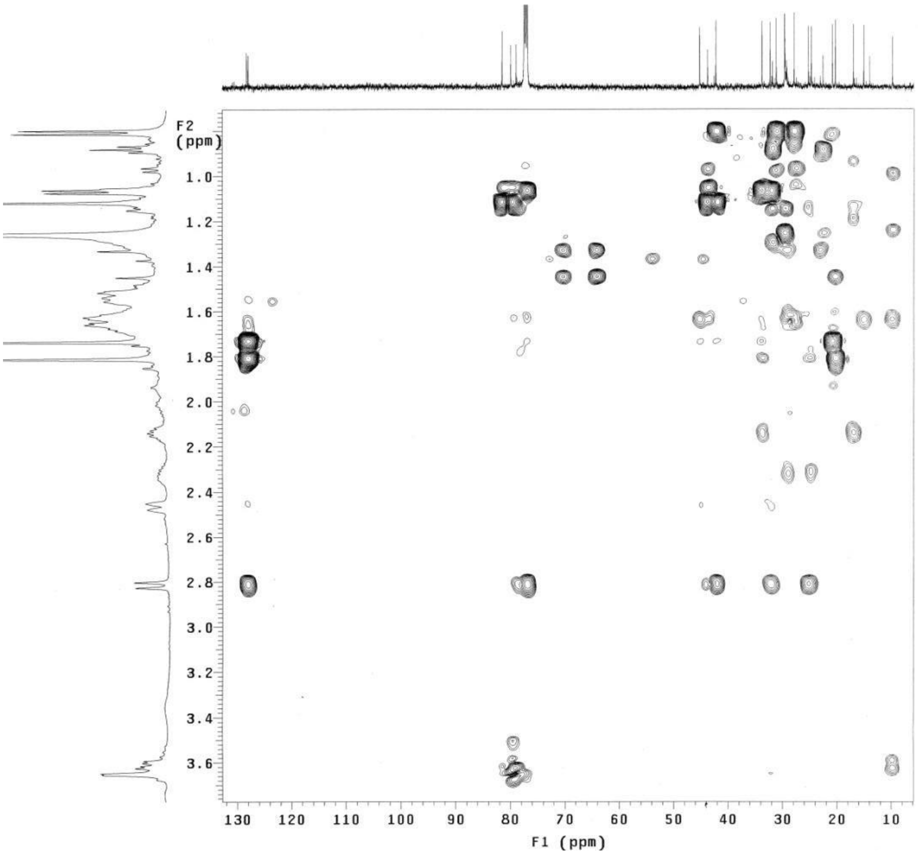

Figure S32. HMBC spectrum of **3** in $CDCl_3$.

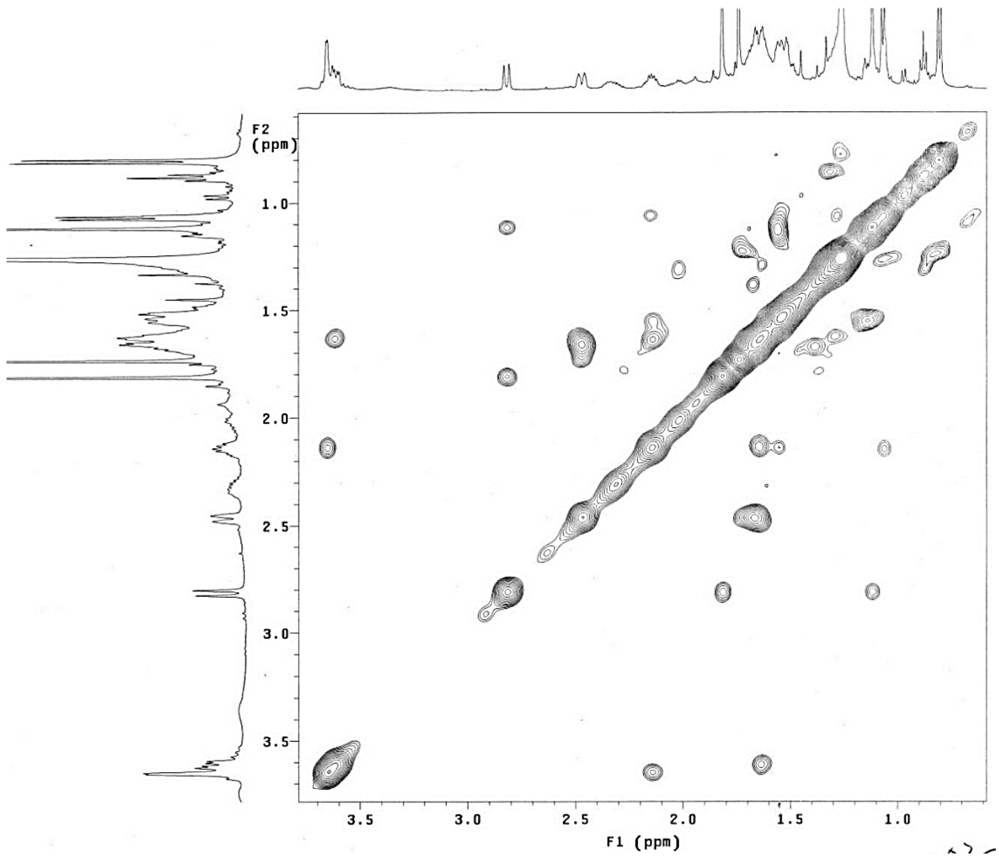

Figure S33. NOESY spectrum of **3** in $CDCl_3$.

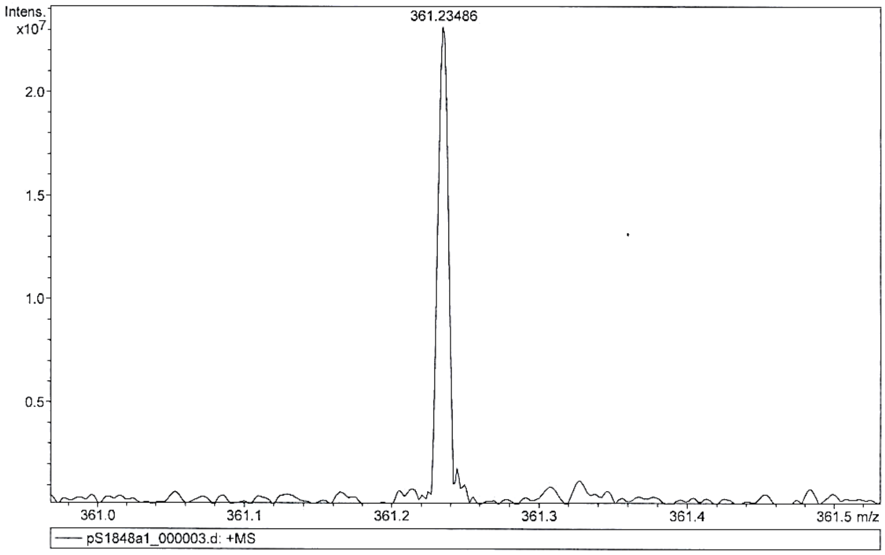

Meas. m/z	#	Formula	Score	m/z	err [mDa]	err [ppm]	mSigma	rdb	e⁻ Conf	N-Rule
361.23486	1	C 20 H 34 Na O 4	100.00	361.23493	0.07	0.20	23.7	3.5	even	ok

Figure S34. HRESIMS spectrum of **4**.

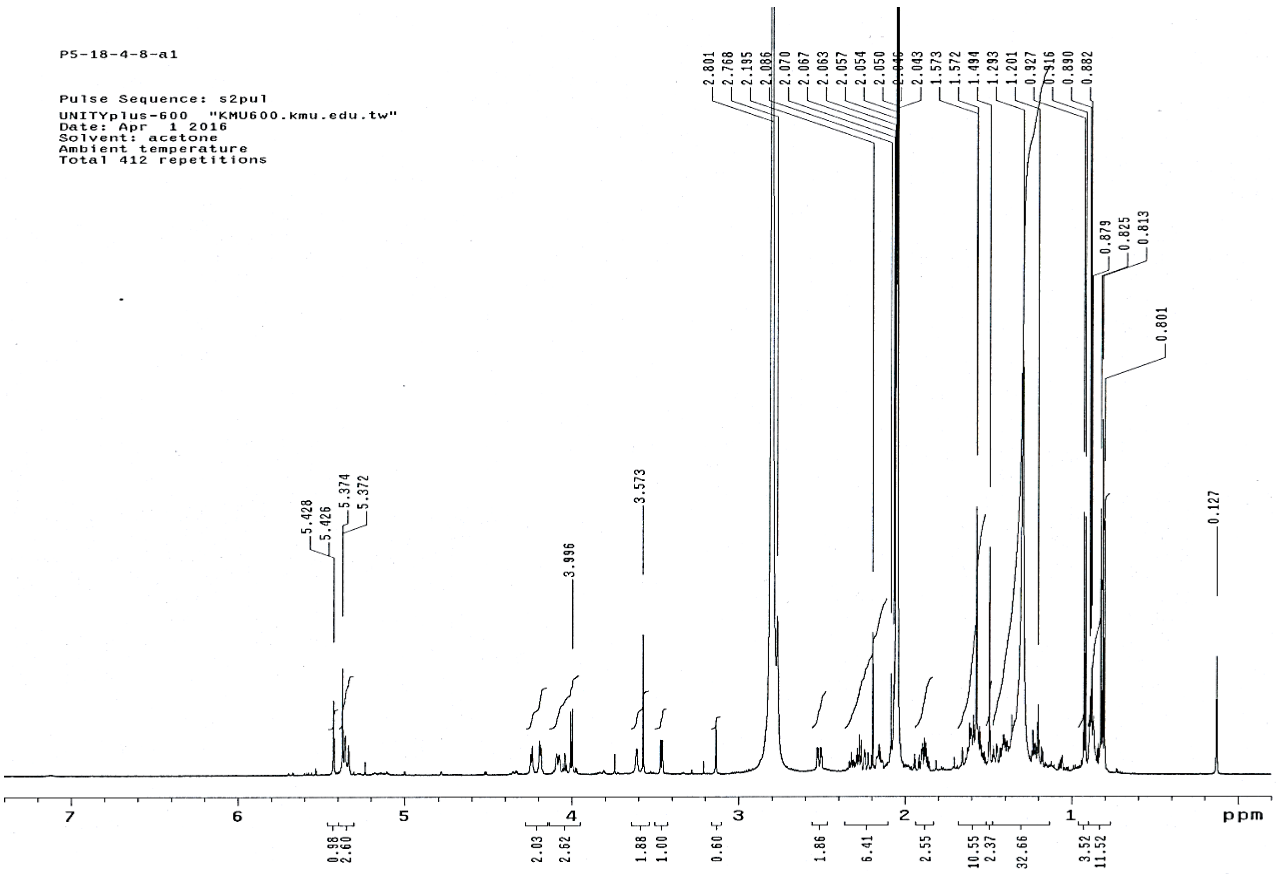

Figure S35. ^{1}H NMR spectrum of **4** in acetone-d_6 at 600 MHz.

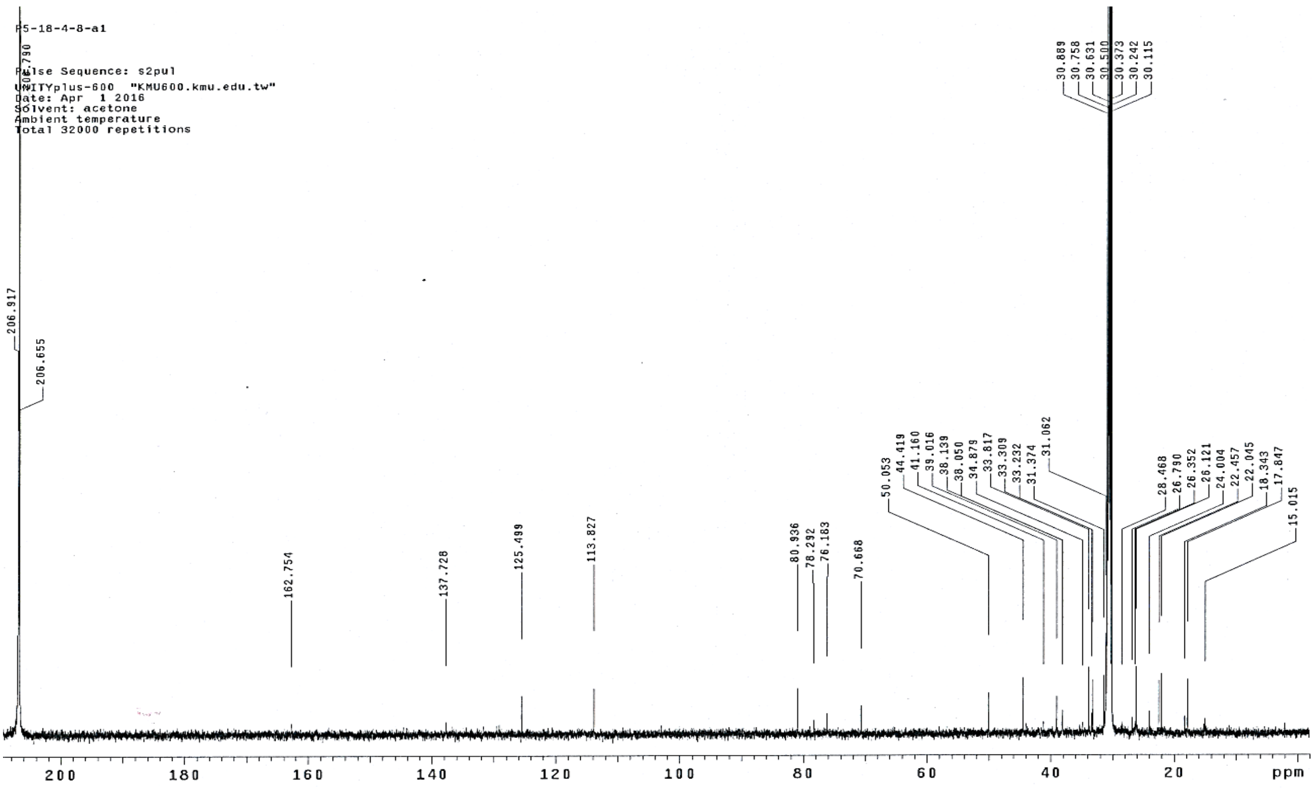

Figure S36. ^{13}C NMR spectrum of **4** in acetone-d_6 at 150 MHz.

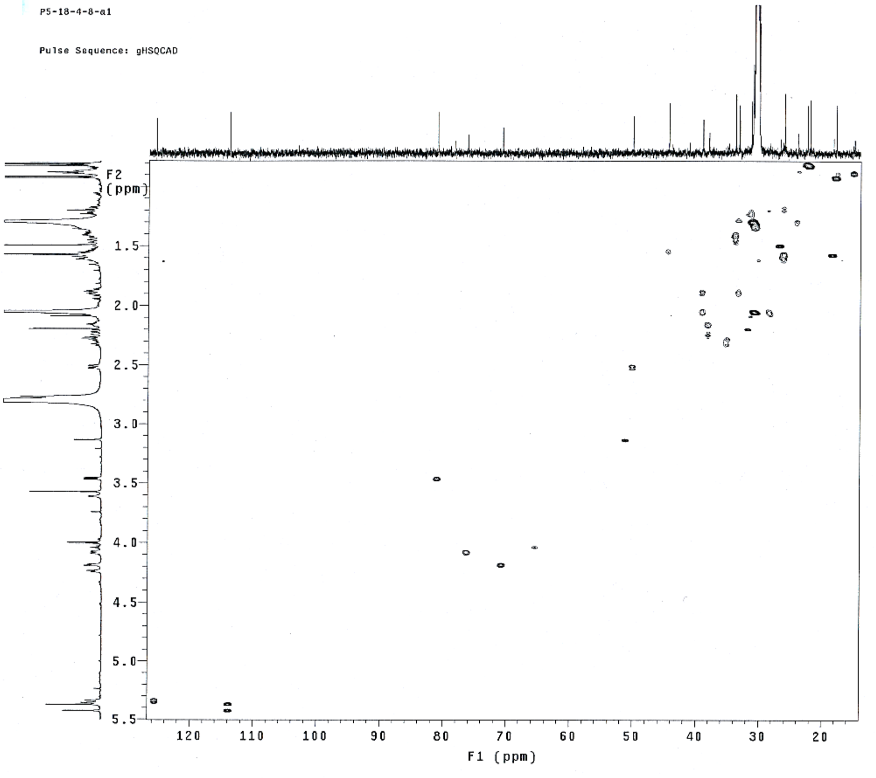

Figure S37. HSQC spectrum of **4** in acetone-d_6.

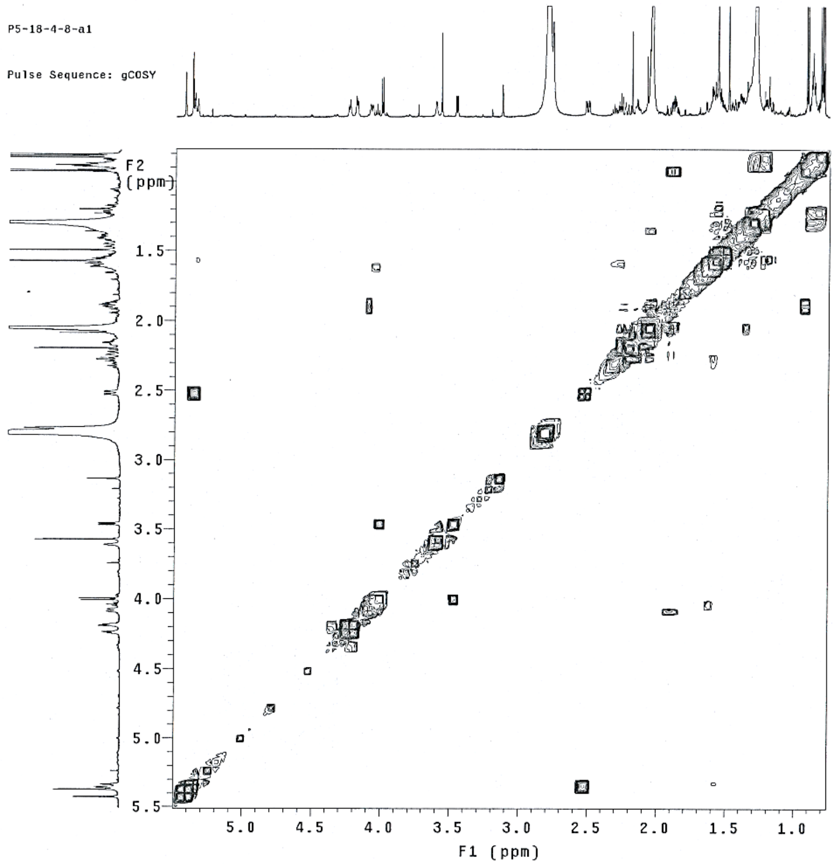

Figure S38. COSY spectrum of **4** in acetone-$d_{6.}$

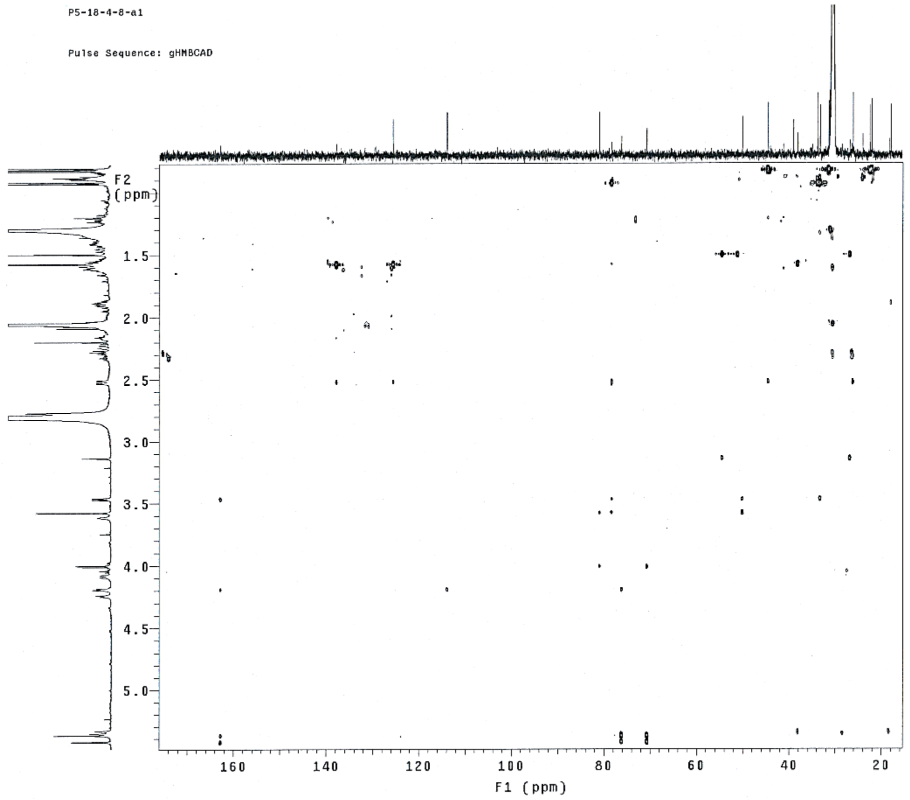

Figure S39. HMBC spectrum of **4** in acetone-d_{6y}.

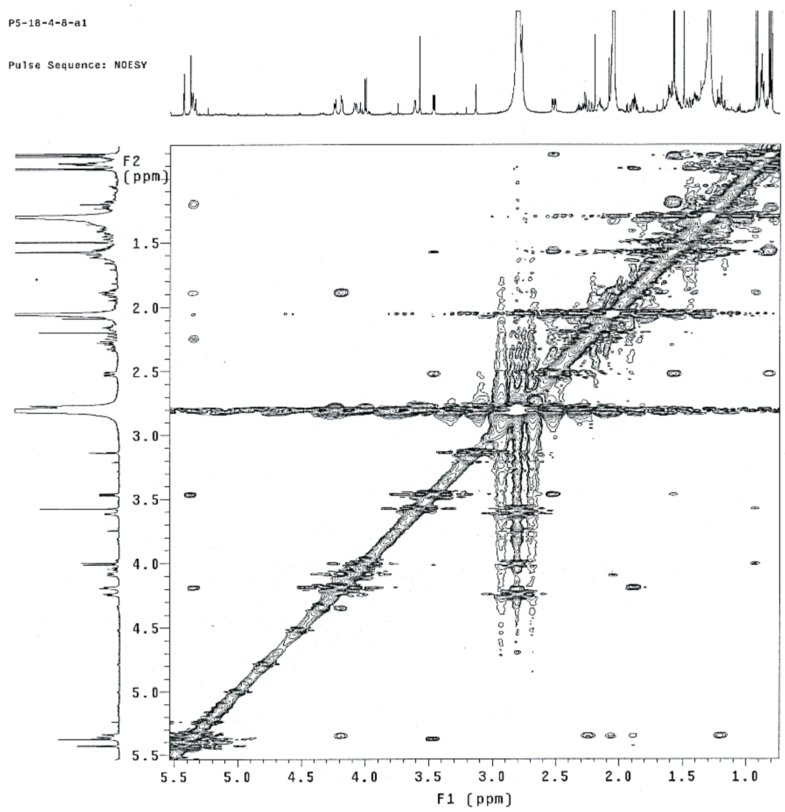

Figure S40. NOESY spectrum of **4** in acetone-d_6.
